# How COVID-19 affected mental well-being: An 11- week trajectories of daily well-being of Koreans amidst COVID-19 by age, gender and region

**DOI:** 10.1371/journal.pone.0250252

**Published:** 2021-04-23

**Authors:** Incheol Choi, Joo Hyun Kim, Namhee Kim, Eunsoo Choi, Jongan Choi, Hye Won Suk, Jinkyung Na

**Affiliations:** 1 Department of Psychology, Seoul National University, Seoul, Republic of Korea; 2 Center for Happiness Studies, Seoul National University, Seoul, Republic of Korea; 3 School of Psychology, Korea University, Seoul, Republic of Korea; 4 Department of Psychology, Kangwon National University, Chuncheon-si, Republic of Korea; 5 Department of Psychology, Sogang University, Seoul, Republic of Korea; University of Toronto, CANADA

## Abstract

The present study examined the daily well-being of Koreans (*n* = 353,340) for 11 weeks during the COVID-19 pandemic (January 20 –April 7). We analyzed whether and how life satisfaction, positive affect, negative affect, and life meaning changed during the outbreak. First, we found that the well-being of Koreans changed daily in a cubic fashion, such that it declined and recovered during the early phase but declined substantially during the later phase (after COVID- 19 was declared world pandemic by WHO). Second, unlike other emotions, boredom displayed a distinctive pattern of linear increase, especially for younger people, suggesting that boredom might be, in part, responsible for their inability to comply with social distancing recommendations. Third, the well-being of older people and males changed less compared to younger people and females. Finally, daily well-being dropped significantly more in the hard-hit regions than in other regions. Implications and limitations are discussed.

## Introduction

The beginning of the year 2020 was marked by the global pandemic of COVID-19. Since the first pneumonia case of unknown cause was reported in Wuhan, China, on November 17, 2019 [1], the virus has been rapidly spreading worldwide with tragic effects. Within six months, more than 10 million people around the world were infected, and about 500,000 people have died [2]. No cure or vaccine for the virus was invented at the time of writing this article. The influence of the COVID-19 pandemic is not limited to physical health. Nearly every aspect of human life has been affected, including the economy, travel, politics, religion, education, and international relations. Individuals’ social lives have also been substantially compromised by the outbreak, as social contacts have been strongly discouraged or even prohibited. All these unexpected and unprecedented life changes and uncertainties lead to a growing concern for the psychological well-being of people [3, 4], calling for empirical studies on the nature and magnitude of the psychological effects of COVID-19.

Some preliminary reports on the effects of COVID-19 on individuals’ well-being have been published [5–7]. For example, researchers showed an increased prevalence of mental problems, such as anxiety and depression, among Chinese during the outbreak [6]. Another study from Italy reported that adults in quarantine complained about various negative emotions, such as boredom [5]. The most recent longitudinal study conducted in China across two time-points (during the initial outbreak and peak of COVID-19 four weeks later) found that a significant number of people reported moderate-to-severe stress, anxiety, and depression [8]. These studies provided some evidence suggesting that the COVID-19 pandemic could have negative effects on individuals’ mental health. However, these studies are too preliminary to deal with the magnitude and pervasiveness of the psychological effects of the COVID-19 due to their lack of diversity in terms of samples and measures. Even the longitudinal study by Wang and colleagues (2020) covered only four weeks after the outbreak with just two time-points. Consequently, it is difficult to estimate the overall trajectory of psychological changes resulting from the COVID-19 outbreak. Therefore, a more comprehensive investigation is needed to address questions like whether COVID-19 affects well-being, how the effects of COVID-19 on well-being vary during the pandemic crisis, what groups are more or less affected (e.g., teens vs. elderly, males vs. females), and what aspects of well-being (e.g., life satisfaction vs. affects) are more vulnerable to the COVID-19 crisis.

To answer the above questions adequately, it is ideal to conduct a study that would incorporate 1) a large sample size with demographic diversity, 2) daily real-time rather than retrospective tracking of well-being, 3) diverse measures of well-being that can uncover various aspects of mental wellness, and 4) a sufficiently long period of observation that includes the beginning, the peak and the end of the outbreak. However, it is almost impossible to design such a study beforehand. Fortunately, the Center for Happiness Studies at Seoul National University (SNU) of Korea, in collaboration with the Kakao, the number one South Korea online platform company, has been conducting a daily online survey on the well-being of Koreans since 2018. About 1.2 million and 1.5 million Koreans participated in the survey, with the average number of daily participants of 2,859 and 3,915 in 2018 and 2019, respectively [9, 10]. Moreover, the survey includes diverse measures of well-being, ranging from life satisfaction [11], positive affect (e.g., joy and calm), negative affect (e.g., anxiety, depression, and boredom) [12], to life meaning [13], as recommended by well-being scholars and international institutions, such as OECD [14] and UN [15].

Furthermore, studying the South Korea case has a couple of additional advantages. First, approximately two-thirds of the total cases surged in the space of only a few days around one cluster from a church in Daegu city in late February [16]. A single spreader known as “patient 31”, who was a congregant at Shincheonji Church of Jesus in Daegu, was in contact with at least 1,160 people attending church services. Therefore, the comparison between Daegu and other regions in South Korea provides a rare natural experimental setting. Second, despite the possibility of a second wave of COVID-19, Korea is one of the few countries to successfully flatten the curve within a relatively short time frame. The number of daily infected patients peaked at 909 (February 29) but gradually decreased down to 50 in early April. Therefore, the SNU / Kakao dataset has many characteristics of an ideal study of the impact of COVID-19 on mental well-being.

To examine well-being trajectories over the course of COVID-19, we included data collected from January 20 to April 07, as these data were available at the time of this study. January 20 was the day when the first confirmed case was announced in South Korea and the level-1 alert was sent out. Within a month, two more alerts were issued, including the highest-level alert right after the patient 31 was confirmed (February 23). Since March, the number of daily confirmed cases in Korea decreased dramatically, which led many health experts and media over the world to claim that Korea successfully flattened the curve [17]. In stark contrast, the global situation got worse, forcing WHO to declare COVID-19 as a pandemic on March 11. In response to the worsening global situation, the Korean government ordered a nationwide intensive social distancing on March 22. Thus, the current analysis could show not only people’s initial psychological responses to the COVID-19 epidemic but also their reaction and (or) adaptation to the recovery of the local situation and the continuing/worsening global situation.

## Methods

### Participants

Individuals ranging in age from 14 to 71 years (*N = 353*,*340*, *M*_age_ = 30.70, *SD*_*age*_ = 12.34) completed an online well-being survey from Kakao company during the study period (January 20 to April 07). Overall, 79% of respondents were female, and 36.8% were in their 20s (Table 1).

**Table 1:** Demographic characteristics of respondents (N = 353,340).

	*n*	%
Gender		
Female	278978	79.0
Male	74362	21.0
Age		
10s	55057	15.6
20s	130133	36.8
30s	85255	24.1
40s	52013	14.7
50s	25242	7.1
Over 60s	5640	1.6
Region		
Daegu/Gyeongbuk	28430	8.0
Others	324910	92.0
Number of responses per person		
1	261869	74.1
2 or more	91471	25.9

*Note*. A total percentage of age is not 100 because of rounding.

The survey was performed on an online survey platform launched by Kakao cooperation, one of the largest Internet companies in South Korea. Any Kakao app users can voluntarily participate in the survey via smartphone or computers (http://together.kakao.com/hello), and they can respond to each survey at any time and across multiple occasions. During the study period, on average, 4,472 respondents participated in the survey per day, and a total of 490,014 responses were used in the analysis. The proportion of respondents across different regions reflected the regional distribution of the South Korean population. The privacy policy of the company restricted the collection of participants’ demographic information to age, gender, and region of residence. Conducting secondary data analysis of the Kakao survey was approved by the Kangwon National University Institutional Review Board (#201910009002).

### Measures

#### Well-being

A 10-item questionnaire was used to measure well-being. The items were adapted from well-established measures of subjective well-being (life satisfaction, positive affects and negative affects) and eudaimonic well-being (life meaning) [18]. Previous literature well addressed that the items selected are valid to represent various aspects of well-being [11–13]. In addition, the 10-item index was also validated among Korean populations [19].

Life satisfaction and life meaning were measured using a single item each, asking about the extent to which respondents were satisfied with their lives and felt that it was meaningful. For affective well-being, participants responded to 8 questions, each question assessing “How much are you feeling each emotion at this moment?” anchored by three positive affects (PA: happy, joyful, and relaxed) and five negative affects (NA: bored, annoyed, anxious, depressed, stress) [19, 20]. Participants responded to each of the well-being indicators on an 11-point scale ranging from 0 to 10 (e.g., 0 = not satisfied, 10 = very satisfied; see S1 Table for the actual questionnaire). The well-being index was created by averaging scores from 10 items (*α* = .913). Composite scores for PA and NA were also computed by averaging across three or five emotions, respectively, with high internal consistency (*α* = .875 for positive affect, *α* = .886 for negative affect).

#### Demographic variables

Gender, birth year, and residential place were self-reported. The age was estimated from the reported birth year, and individuals were divided into three age groups, young (10s-20s), middle (30s-40s), and old (50s-70s), in the main analysis.

## Results

Considering the large sample size, we adopted a more conservative approach by performing tests at a significance level of *α* = .001, as in Stone et al. [21].

### Analytic strategies

Polynomial models were used to examine the well-being trajectories over 79 days. More specifically, we fitted polynomial models in the multilevel modeling framework using the *lme4* package in *R* [22], considering the nested structure of the data. About 26 percent of participants answered the survey more than once during the study period. The demographic characteristics, such as age, gender, and region of residence, were included in the model to examine individual differences in the trajectories.

Ten-fold cross-validation [23] was used to select the optimal degree of a polynomial for each well-being measure. For the 10-fold cross-validation, we split the data into 10 roughly equal-sized subsets. For each measure, we fitted polynomial models with varying degrees (from 0 to 6) to nine subsets of the data. We obtained the prediction error of the estimated model by calculating the squared difference between the observed score and the predicted score for the fresh data that was set aside in estimation. This process was repeated 10 times by using each subset of the data that was set aside to calculate the prediction error. Finally, the mean predictor error was calculated for the entire data. For each well-being measure, we examined the mean predictor errors across different degrees of polynomials. Due to the large sample size, the prediction error kept decreasing as the degree of polynomial increased. To avoid overfitting, we chose the degree at an elbow point, where the prediction errors started to stabilize, as the optimal one for further analyses. Based on the cross-validation results, we chose a linear model for boredom and joy and a cubic model for all the other well-being measures (S1 Fig). Using the respective model, we analyzed not only the overall well-being index but also each subcomponent of the index. In the following section, we first report the results based on the well-being index and then describe additional results from subcomponents we thought were interesting as well as informative. We also note that detailed results regarding all subcomponents of the well-being index are reported in the Supporting information.

### Well-being changes during COVID-19 outbreak

**Fig 1:**
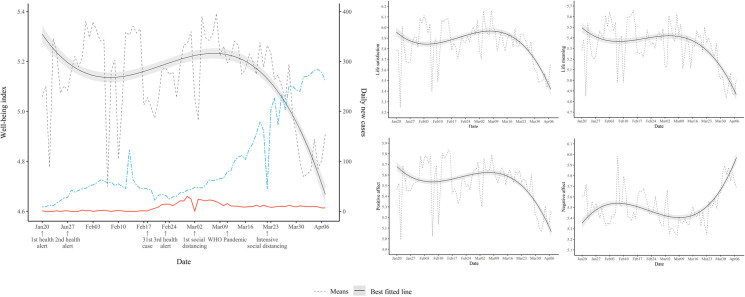
Estimated trajectories with 99.9% confidence intervals of five well-being measures (well-being index, life satisfaction, life meaning, PA, and NA). Note. Dashed grey line indicates the estimated daily means of each well-being measure. The red and blue dashed line represents square rooted daily new cases of South Korea and Worldwide, respectively.

Our first question was whether/how the daily well-being of Koreans changed during the COVID-19 outbreak. As can be seen in Fig 1, overall well-being displayed the decline–recovery–decline pattern, hence a cubic pattern (S2 Table). A close look at Fig 1 shows that the overall well-being declined up to February 10, then gradually ascended to the first week of March, and steeply declined after that. Recall that WHO declared COVID-19 a pandemic on March 11 and the Korean governement implemented the intensive social distancing policy on March 22. Interestingly, the initial decline in well-being occurred well before the situation in Korea worsened, consistent with the past research showing that well-being starts to decline before the critical event, such as divorce and unemployment [24]. It is even more intriguing that the well-being of Koreans declined very steeply after the first week of March, during which the situation in Korea started to improve. We discuss later why this might have been so. The same cubic pattern of well-being change emerged for life satisfaction, PA, NA, and life meaning (Fig 1 and S2 Table).

### Trajectories of emotions

**Fig 2:**
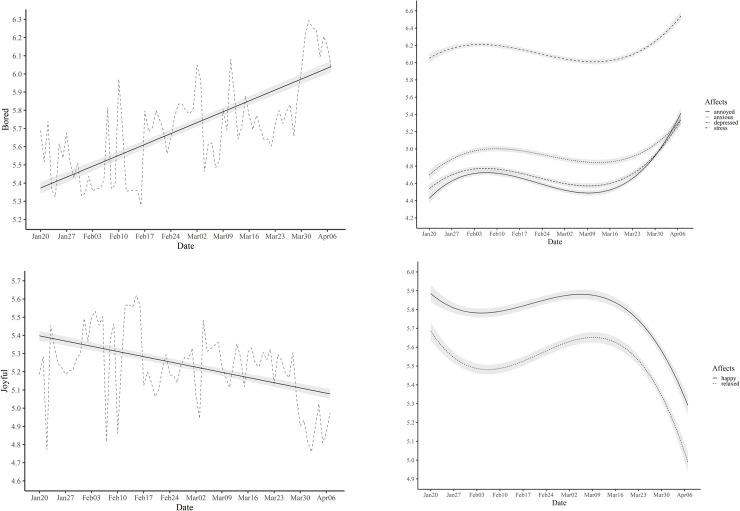
Estimated trajectories with 99.9% confidence intervals of negative affect measures (bored, annoyed, depressed, anxious, and stress) and positive affect measures (joyful, happy, and relaxed). Note. Dashed grey line indicates the estimated daily means of boredom and joy.

To examine whether the change patterns varied by different emotions, we analyzed each emotion measure separately. As Fig 2 shows, all emotions displayed the identical cubic pattern of change except for boredom and joy (see S3 Table). Specifically, boredom continued to increase, whereas joy continued to decrease during the study period. All the other emotions showed a brief period of recovery in the middle, yet boredom and joy did not. The distinctive pattern of boredom and joy seems very important because it may, at least partially, account for the difficulty people feel in observing social distancing [25]. Social distancing has been promoted as one of the most effective preventive measures against the spread of COVID-19 by health experts. Yet, many people do not seem to follow the advice, potentially costing the lives of others and their own [26]. We speculate that the high level of boredom and lack of joy may make social distancing difficult. Past research has shown that people who are ill-equipped to tolerate boredom willingly inflict electric shock to themselves to fight boredom [27].

**Fig 3:**
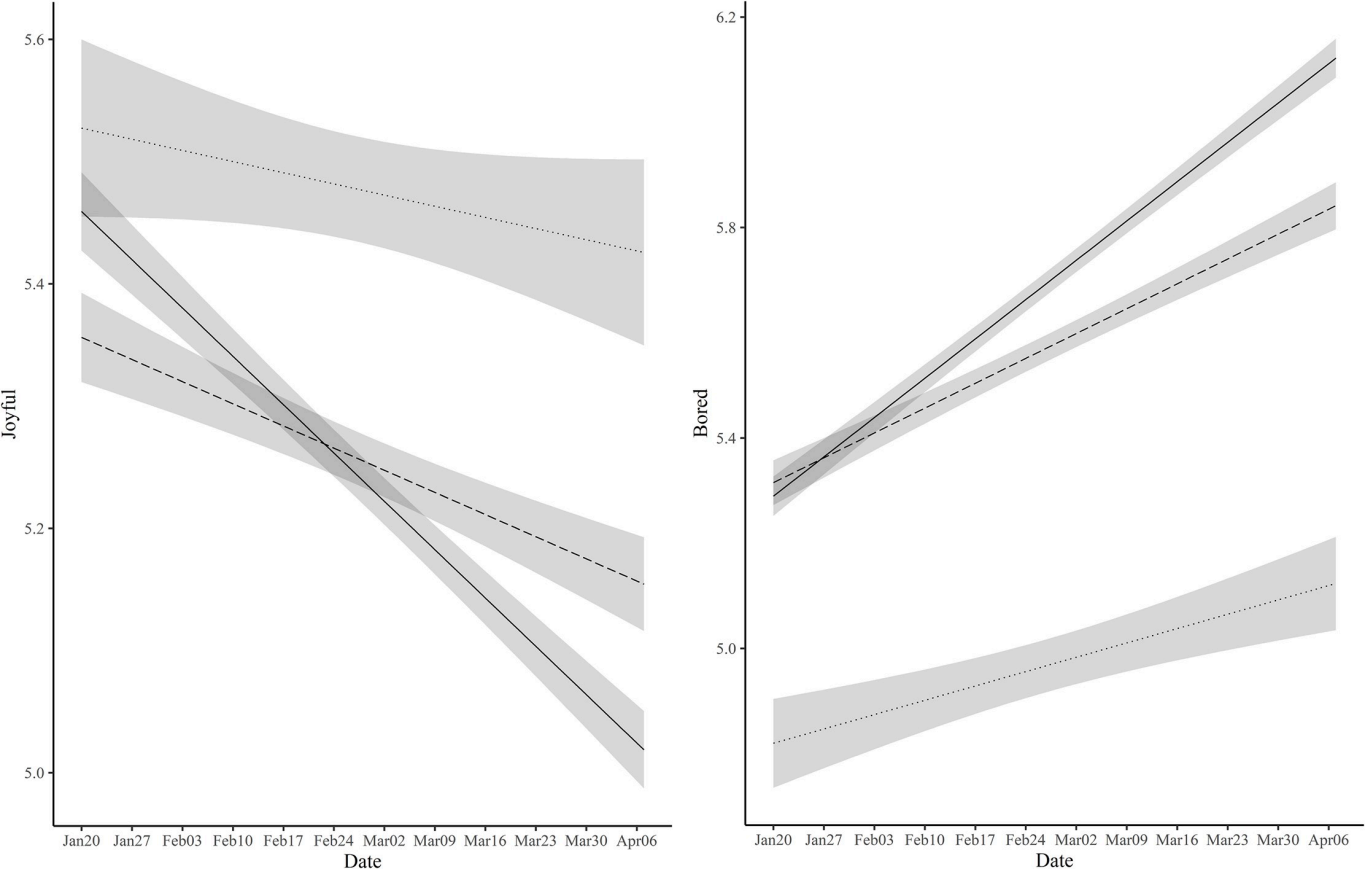
Estimated trajectories with 99.9% confidence intervals of boredom and joy for young, middle, and old groups. Note. Solid line indicates the young group. The dashed and dotted line represents middle and old group, respectively.

The unique pattern of boredom and joy raises the question of who might be suffering from boredom and joylessness most. The frequent media reports [28], as well as our observations of young people failing at social distancing, imply that young people suffered from boredom and joylessness most. To answer the question, we tested whether the model with interactions between the polynomials and the age groups yielded a significantly better fit compared to the baseline model without the interactions. The results showed that the model fit significantly increased after adding the interaction terms for boredom and joy, *χ*^2^(2) = 197.75, *p* < .001 for boredom, *χ*^2^(2) = 135.65, *p* < .001 for joy (S4 Table). As can be seen in Fig 3 and S5 and S6 Tables, younger people (10s-20s) manifested more pronounced change compared to older people in terms of the surge of boredom (*b =* .833, *SE* = .018, *p* < .001 for young, *b =* .527, *SE* = .022, *p* < .001 for middle, *b =* .304, *SE* = .043, *p* < .001 for old) and the falling of joy (*b =* -.441, *SE* = .016, *p* < .001 for young, *b =* -.202, *SE* = .019, *p* < .001 for middle, *b =* -.102, *SE* = .037, *p* = .006 for old).

### Regional comparison: Hard-hit regions vs. others

**Fig 4:**
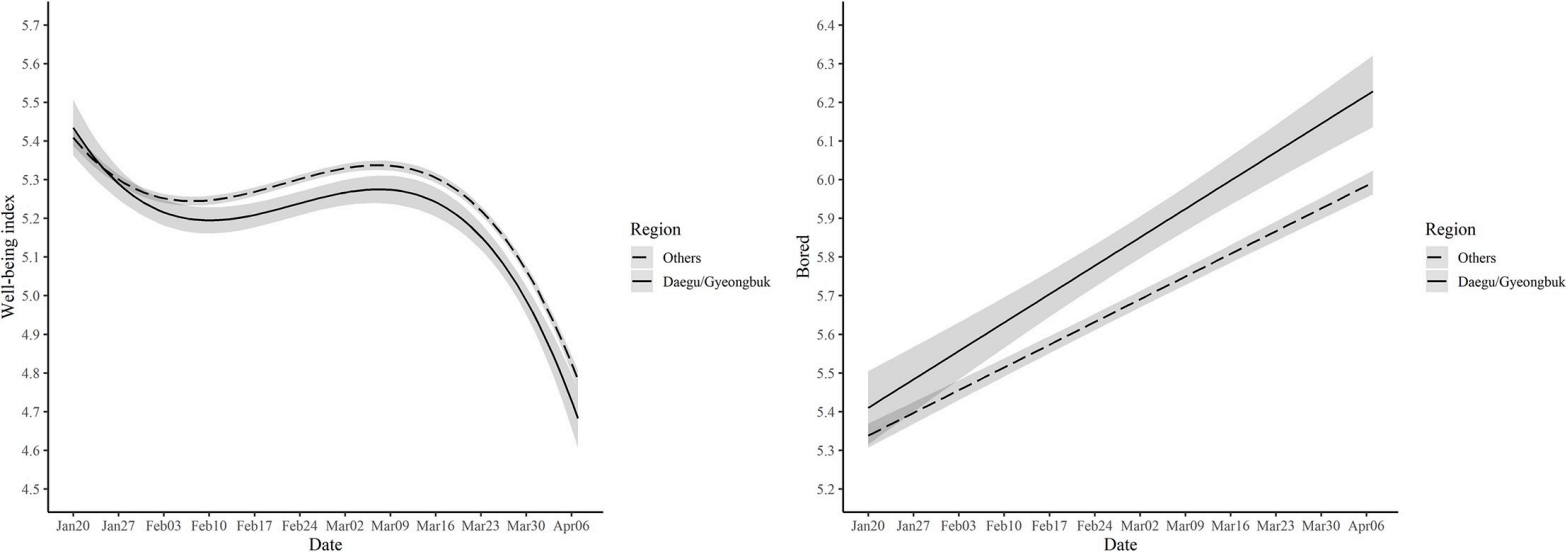
Estimated trajectories of well-being index and boredom for Daegu/Gyeongbuk and other regions. Note. 95% confidence interval is displayed for the well-being index, while 99.9% confidence interval is used for boredom.

As stated earlier, a comparison between the city of Daegu (and its adjacent areas, Gyeongbuk province) with the rest of Korea offers a rare opportunity for a natural experiment. Note that about two-thirds of patients in Korea during this period were residents of Daegu/Gyeongbuk. First, we examined the mean differences in well-being between Daegu/Gyeongbuk and other regions during the study period controlling for age and gender. As can be seen in S7 Table, Daegu/Gyeongbuk scored lower compared to the other regions in most well-being measures. For example, the estimated regional trajectories in Fig 4 show that the Daegu/Gyeongbuk residents tended to manifest a lower level of overall well-being throughout the study period, except for the first week. Second, we further examined whether the changing pattern differed between the two regional groups by comparing an interaction model with a baseline model without interaction. For the overall well-being index, the interaction model did not significantly improve the model fit compared to the baseline model, *χ*^2^(3) = 7.318, *p* = .062, suggesting that the shape of the trajectory did not differ between the two regions (S8 and S9 Tables). However, in the case of boredom, a model with the interaction was better compared to the baseline model without the interaction, *χ*^2^(1) = 11.464, *p* = .001 (S8 and S10 Tables). As can be seen in Fig 4, the boredom level in Daegu/Gyeongbuk region (*b =* .818, *SE* = .046, *p* < .001) increased more steeply compared to the other regions (*b =* .654, *SE* = .014, *p* < .001). This is not surprising given that social distancing was most strongly enforced in Daegu/Gyeongbuk. Our regional contrast suggests that the changes in the daily well-being of Koreans during the study period were partially, if not entirely, due to COVID-19.

### Age differences

As virus, COVID-19 is more fatal to people older than 60 years of age compared to younger people [29, 30]. Accordingly, are psychological perils also greater for older than for younger people? Alternatively, since older people are more emotionally stable and better equipped to deal with existential threats [31, 32], do older people suffer less mentally?

**Fig 5:**
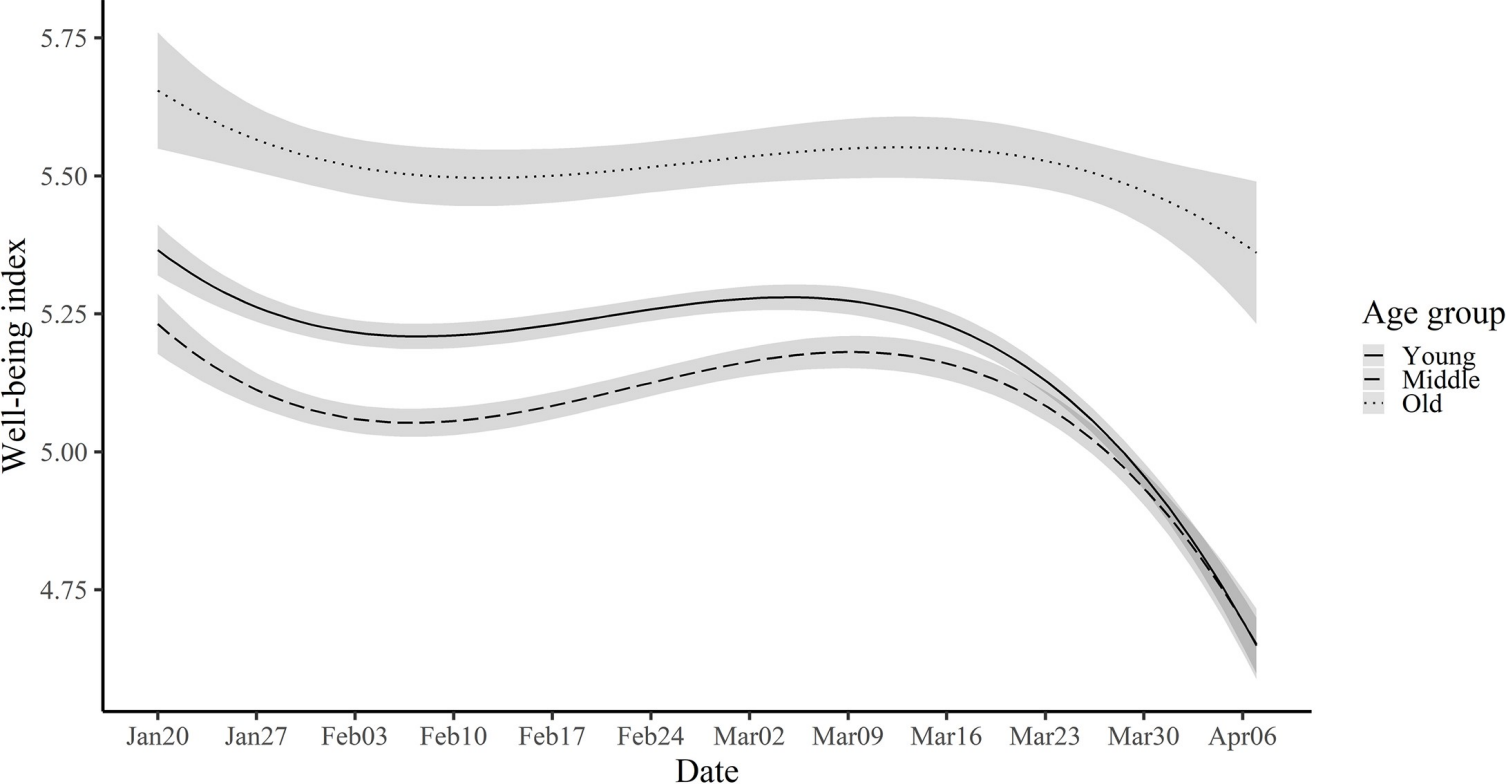
Estimated trajectories of well-being index for young, middle, and old groups.

**Fig 6:**
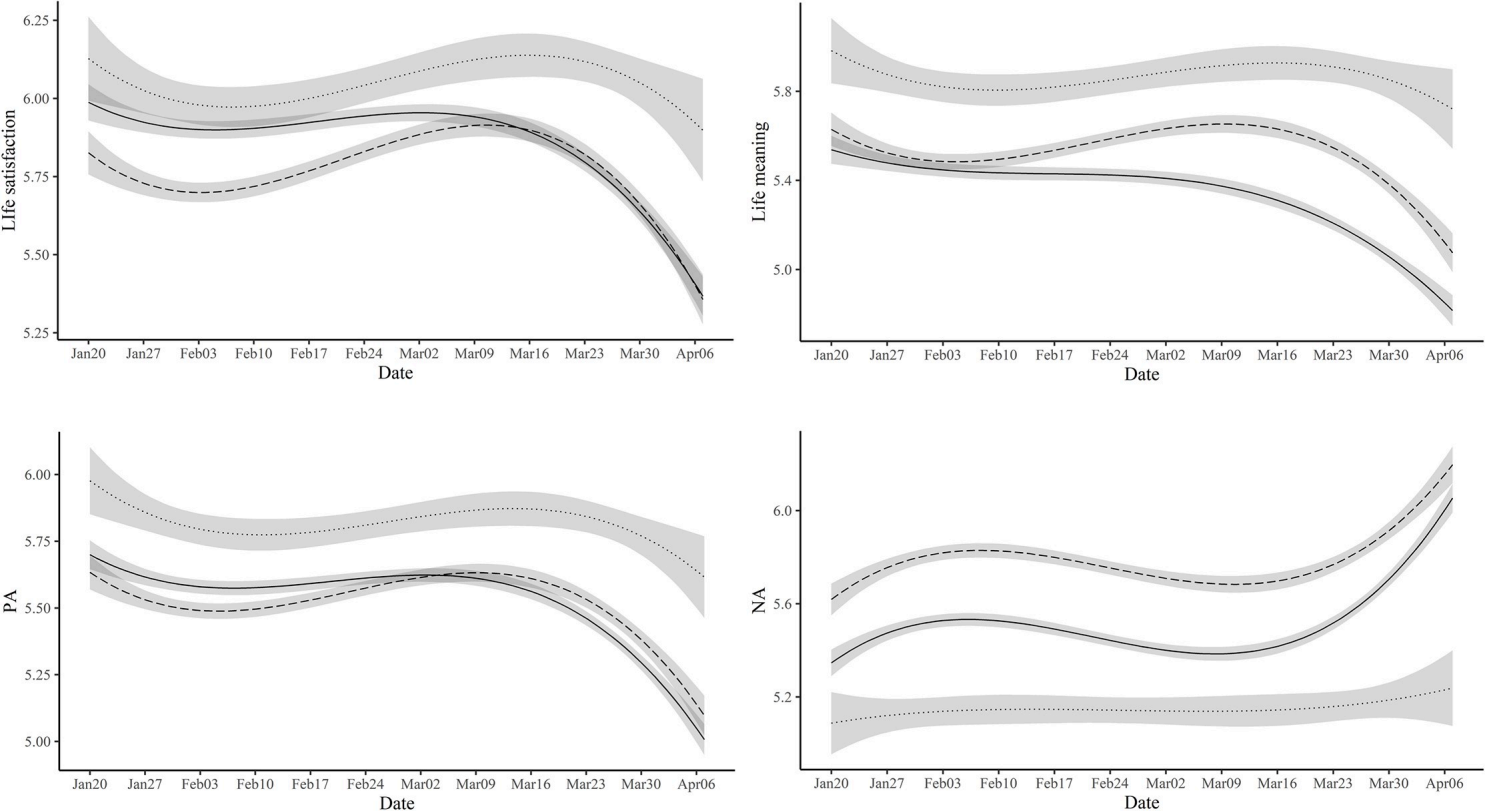
Estimated trajectories of life satisfaction, life meaning, PA, and NA with 99.9% confidence intervals for young, middle, and old groups. Note. Solid line indicates the young group. The dashed and dotted line represents middle and old group, respectively.

To answer these questions, we compared three age groups: Young (10s & 20s), Middle (30s & 40s), and Older (over 50s). First, as shown in S7 Table, the well-being index showed a U-shape. Overall, it was the highest among older adults and lowest among middle-aged adults, which is consistent with the baseline well-being differences among Koreans across different age groups [9]. Next, we tested whether the trajectories of well-being measures varied across different age groups by comparing model fit, as in the previous section. The results showed that the model fit significantly increased after adding the interaction term for the well-being index, *χ*^2^ (6) = 195.35, *p* < .001 (S4 Table). As Fig 5 indicates, overall, old people showed the highest level of well-being, which was followed by young people, and then middle-aged people. All the age groups displayed a pattern of decline–recovery–decline. However, toward the end of the study period, young and middle-aged groups reached a decreased well-being of similar levels, yielding an increased gap from old people. The same pattern emerged for life satisfaction, NA, and life meaning (Fig 6 and S12 Table), except that middle-aged people showed a higher level of life meaning than young people. For PA, the pattern appeared to be similar as other well-being indices, but it was not statistically significant.

**Fig 7:**
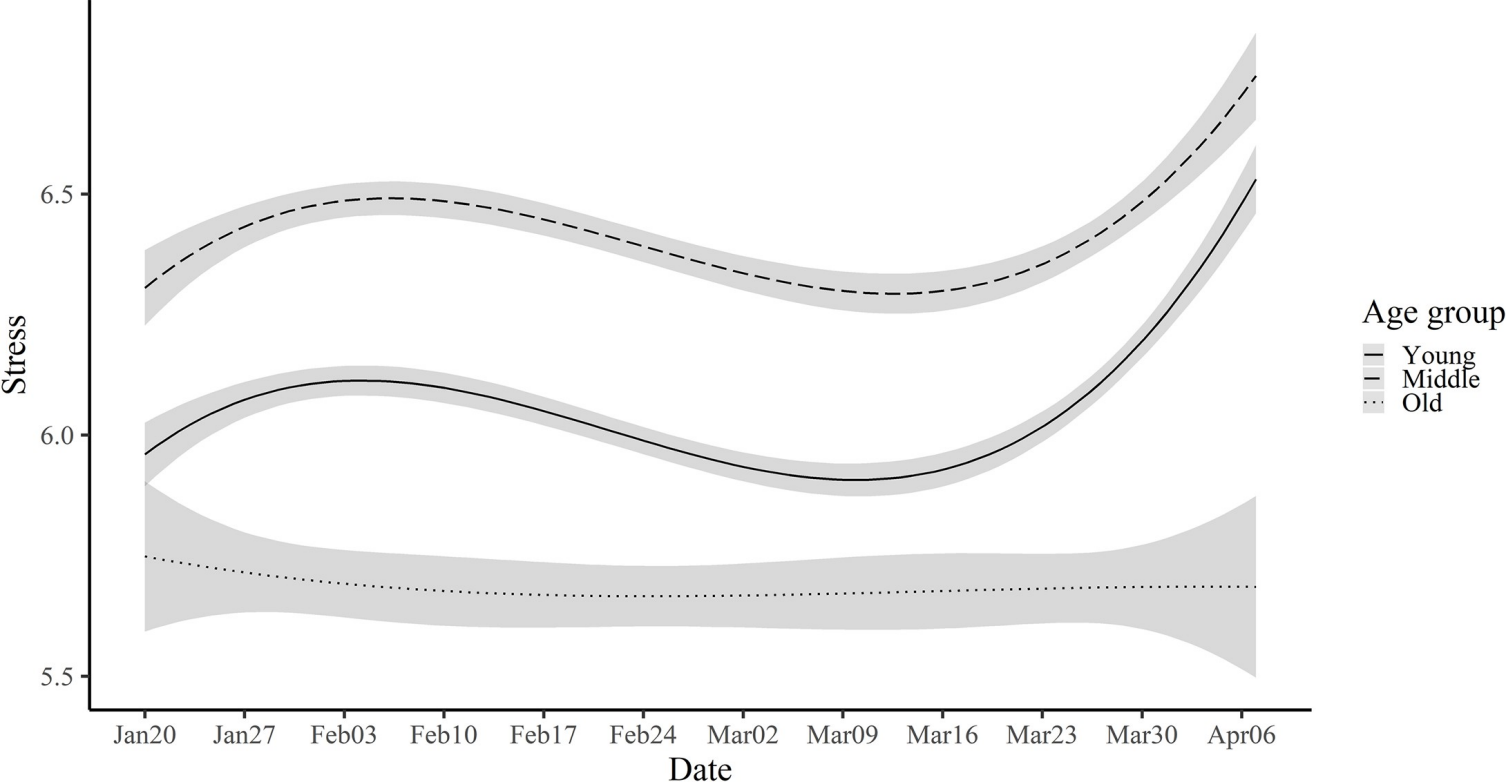
Estimated trajectories of stress with 99.9% confidence intervals for young, middle, and old groups.

As reported earlier, age differences were also observed for specific emotions. In general, the gap between old people and middle-aged/young people tended to increase toward the end of the study period as illustrated in the stress trajectories. (Fig 7 and S5 Table).

It is intriguing that despite greater biological vulnerability to COVID-19, older people were more resilient than younger people in terms of emotional well-being. The socioemotional selectivity theory [33] posits that with aging, people voluntarily narrow their social network and limit their activities to those they enjoy; hence, social distancing might be a more natural process for them. Therefore, the social lives of older people might have been less disrupted by COVID-19. Alternatively, older people might simply be better able to cope with the fear of death compared to their younger counterparts. Future research should uncover the exact mechanism(s) why older people’s mental well-being is less affected by COVID-19 than that of younger people.

### Gender differences

**Fig 8:**
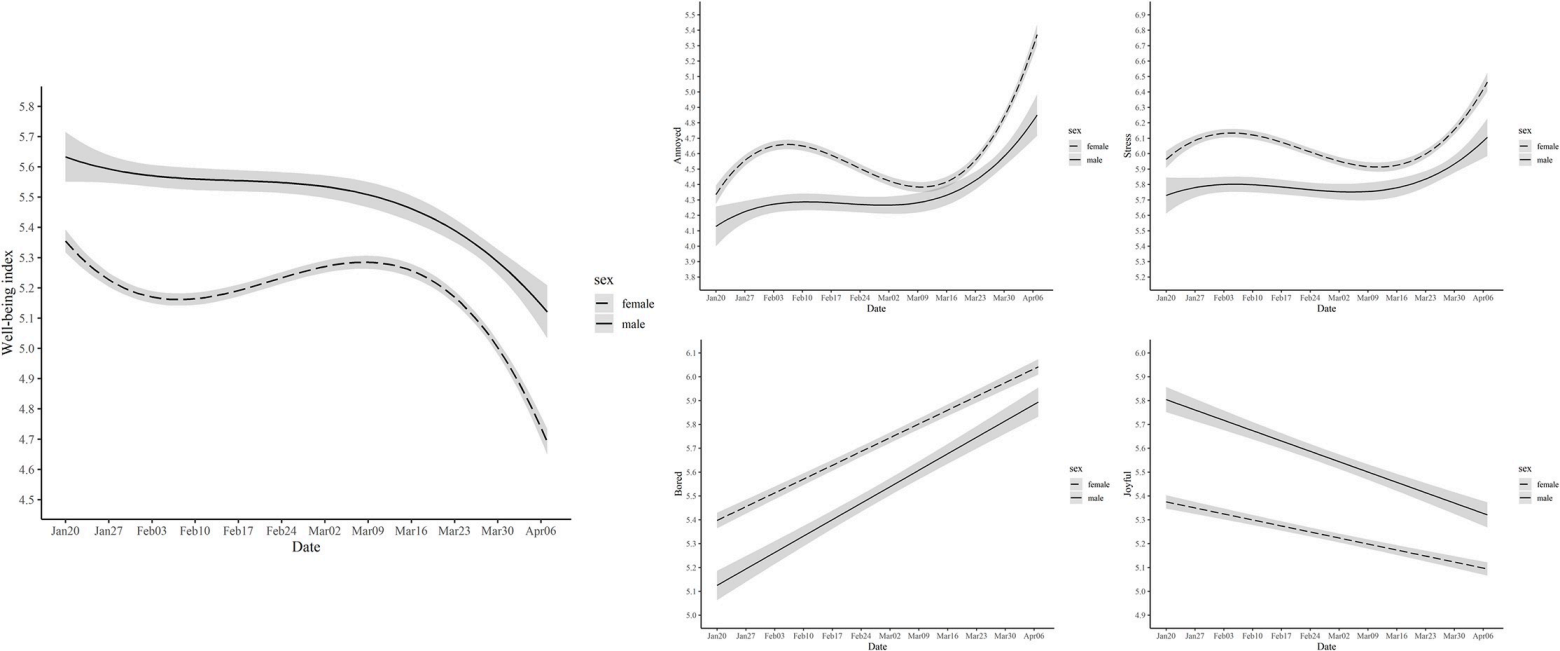
Estimated trajectories of well-being measures with 99.9% confidence intervals for male and female.

Whose well-being was affected more by COVID-19, males and females? First, as shown in S7 Table, the well-being of women was lower compared to that of men during the study period across all the measures. However, the gender differences were consistently observed before COVID-19 [9, 10], suggesting that they may reflect the baseline gender differences in well-being among Koreans, rather than differences related to COVID-19. Hence, to further explore COVID-19 related gender differences, we tested whether changes in daily well-being would show different patterns for men and women. As in previous sections, we compared an interaction model with a baseline model without interactions. Gender difference in change patterns was significant for all measures (S13 Table). As can be seen in Fig 8, women tended to experience a greater amount of overall change than men despite some fluctuations in the middle. This was true for most of the specific emotions (see Fig 8 for annoyance and stress as examples). Interestingly, however, the slope for boredom and joy was steeper for men than for women (Boredom: *b* = .769, *SE* = .030, *p* < .001 for male vs. *b* = .644, *SE* = .015, *p* < .001 for female; Joy: *b* = -.483, *SE* = .026, *p* < .001 for male vs. *b* = -.281, *SE* = .013, *p* < .001 for female). In other words, on average, men experienced a larger change in boredom and joy compared to women, unlike other well-being indices (see S15 and S16 Tables for details).

Our dataset did not distinguish homemakers from working moms, singles from married individuals, and families with children from those without children. Hence, more fine-grained analyses concerning gender differences in well-being during the outbreak should be conducted in future studies.

## Discussion

This study is one of the first large-scale observational studies that examined the real-time self-reports of different aspects of well-being in response to living during an unprecedented epidemic of COVID-19. We examined trajectories of well-being during the first 11 weeks of the COVID-19 outbreak in South Korea. Enough data points recorded each day during the pandemic allowed us to capture the change patterns of various aspects of well-being as the pathogen threat unfolded over time.

Our data revealed several interesting as well as important observations. First, COVID-19 significantly affected the well-being of Koreans. The levels of life satisfaction, life meaning, positive affect, and negative affect systematically changed during the study period. Well-being steadily decreased since the first national health alert. Interestingly, for most well-being measures, the decrease was already evident right after the 1^st^ health alert (i.e., when the first confirmed case was announced), reaching the lowest point before the surge of the widespread COVID-19 in Korea. Although well-being bounced back early in March, the recovery did not last but dropped even more substantially from around the time when WHO declared a pandemic and the social distancing was implemented, showing cubic patterns overall. Notably, well-being was significantly lower in Daegu/Gyeongbuk area, the hardest-hit region in Korea, compared to other regions, suggesting that the changes in well-being during the study period were due, at least partially, to COVID-19.

Second, the decline in well-being was much steeper at the end of March. This is very intriguing because in late March and into April, the situation in Korea improved dramatically. The number of daily new patients dropped below 10 during this period. Several reasons could have contributed to this surprising decline. It may reflect psychological reactions to the social consequences of COVID-19, specifically social distancing. The Korean government announced the first social distancing campaign on March 2 and advised the highest level of social distancing on March 22. A major decline in social life during this period might have contributed to a significant decrease in well-being. Or the continuous exposure to news covering COVID-19 pandemic in other countries may have increased fear and uncertainty regarding COVID-19, which in turn may have aggravated Koreans’ well-being. Or reduced income and fear of unemployment started to affect Koreans’ well-being. We speculated that the decline was due to such multiple causes.

Third, patterns of trajectories varied depending on emotions. Unlike other emotions, boredom and joy linearly increased and decreased, respectively. Interestingly, the linear changes in boredom and joy were most evident among younger people (vs. middle-aged and older adults) and males (vs. females). Similar to our findings, preliminary research conducted in other countries revealed that younger adults in Italy suffered from boredom more compared to other age groups [5]. Furthermore, Chinese males, compared to females, were more likely to visit crowded places during the COVID-19 pandemic [34]. Indeed, research on the individual difference in boredom has reported that age and being female are negatively associated with boredom proneness [35, 36].

Taken together, these observations could have important implications not only for researchers in the relevant fields but also for policy makers. First, two different patterns were identified across different emotions, a linear pattern for boredom/joy and a cubic pattern for the other emotions (e.g., anxiety). The finding may imply that a pandemic such as COVID-19 may affect our mental well-being through two pathways, the biological and the social pathway. Specifically, the former may be associated with fear and anxiety of infection and death, while the latter with boredom from a monotonous life. The continuously increasing trend of boredom during the outbreak suggests that one serious obstacle to implementing social distancing or home confinement may be the experience of boredom. Second, we observed significant differences as a function of age and gender in boredom. Boredom, then, from a policy perspective, deserves special attention as a potential threat to pandemic containment measures. In particular, it may be important for policy makers to target these specific groups and develop interventions that help them manage boredom while complying with social distancing. Finally, the present analysis revealed that women showed greater disturbances in well-being than men, which is consistent with the mounting evidence of gender inequality during the COVID-19 pandemic worldwide [37, 38]. As school and daycare closes, the responsibility of taking care of children and unpaid domestic work fell disproportionately on women, resulting in greater strain in their daily lives [39]. Thus, it would be crucial for policy makers to strive to mitigate the negative impacts of COVID-19 on women, especially mothers with young children.

The present research is one of the first nationwide reports to describe the well-being consequences of COVID-19. The various features of the present research (e.g., large sample size, diverse well-being measures, daily well-being tracking over 11 weeks, and multilevel analysis) make the present research unique. Prior observational studies used Twitter or Google Trends as a data source to track well-being or emotional responses to external natural events [40–42]. However, such data are only a proxy for well-being as they often contain irrelevant information (i.e., chatter). In addition, the online data collection used in the present study is superior to most prior studies on collective responses to major life events that relied on retrospective reports [43–45].

Despite these merits, the present research also has some (unavoidable) limitations that make us hesitant to generalize the current findings to other nations. First, COVID-19 is far from over yet. Hence, the current findings might be true only for an earlier phase of the outbreak. Continuing efforts are hence needed to monitor the well-being of citizens until the COVID-19 outbreak finally ends. Second, countries differ vastly in terms of various aspects of the COVID-19 outbreak (e.g., numbers of patients and death, social distancing policies, etc.). Every country should conduct its analysis. We cannot generalize our findings to all other nations. Rather, the present study wishes to share some lessons from Korea’s experience so that we may collectively understand the psychological aftermath of this unprecedented threat and better prepare ourselves for similar future outbreaks. Third, our survey was mainly cross-sectional, although some participants (25.9%) reported their daily well-being more than once during the outbreak, leading us to use multilevel modeling. Robustness checks were conducted to test whether our main findings would hold for different subsamples who provided more than two responses. Results revealed that the main findings of decline-recovery-decline pattern held across these subsamples (see S2 Fig and S17 Table). Hence, it did not capture much within-person variation in well-being. Similarly, the present research adopted a quantitative approach based on a self-reported scale and thus, it would be a worthy endeavor for future research to explore the precise nature of one’s experiences during the pandemic with a qualitative approach. Finally, despite the large sample size, the participants were predominantly young and female, thus, the moderating role of age or gender should be interpreted with caution. We are well aware of this limitation, but we would like to point out that it would be unrealistic to design a true panel study beforehand in cases like COVID-19 and other unexpected disasters.

## Supporting information

S1 Fig Results of the 10-fold cross-validation used to choose the optimal degree of polynomials for each measure. (DOCX)

S2 Fig Results of the ten-fold cross-validation for the well-being index and the well-being trajectory for subsamples providing two or more daily well-being responses. (DOCX)

S1 Table Questionnaire items for the well-being index. (DOCX)

S2 Table Results for the multilevel analyses on the well-being variables including well-being index, positive affect (PA), negative affect (NA), life satisfaction, and life meaning. (DOCX)

S3 Table Results for the multilevel analyses on the positive and negative emotion variables. (DOCX)

S4 Table The results for model comparison between a baseline model and a day by age interaction model for each well-being measure. (DOCX)

S5 Table Results for examining day by age interaction on negative emotion measures. (DOCX)

S6 Table Results for examining day by age interaction on positive emotion measures. (DOCX)

S7 Table Results for multilevel analyses examining the effects of individual characteristics on all well-being measures. (DOCX)

S8 Table The results for model comparison between a baseline model and a day by region interaction model for each well-being measure. (DOCX)

S9 Table Results for examining day by region interaction on well-being measures including well-being index, positive affect (PA), negative affect (NA), life satisfaction, and life meaning. (DOCX)

S10 Table Results for examining day by region interaction on negative emotion measures. (DOCX)

S11 Table Results for examining day by region interaction on positive emotion measures. (DOCX)

S12 Table Results for examining day by age interaction on various well-being measures including well-being index, positive affect (PA), negative affect (NA), life satisfaction, and life meaning. (DOCX)

S13 Table The results for model comparison between a baseline model and a day by gender interaction model for each well-being measure. (DOCX)

S14 Table Results for examining day by gender interaction on various well-being measures including well-being index, positive affect (PA), negative affect (NA), life satisfaction, and life meaning. (DOCX)

S15 Table Results for examining day by gender interaction on negative emotion measures. (DOCX)

S16 Table Results for examining day by gender interaction on positive emotion measures. (DOCX)

S17 Table Results for the multilevel analyses on the well-being index using subsamples providing two or more daily well-being responses. (DOCX)
