# Supplementary material for: How COVID-19 affected mental well-being: An 11- week trajectories of daily well-being of Koreans amidst COVID-19 by age, gender and region
Source: PLoS One. 2021 Apr 23;16(4):e0250252. doi: 10.1371/journal.pone.0250252 (PMC8064534; doi:10.1371/journal.pone.0250252)
Supplement: S1 Fig — (DOCX) [file pone.0250252.s001.docx]

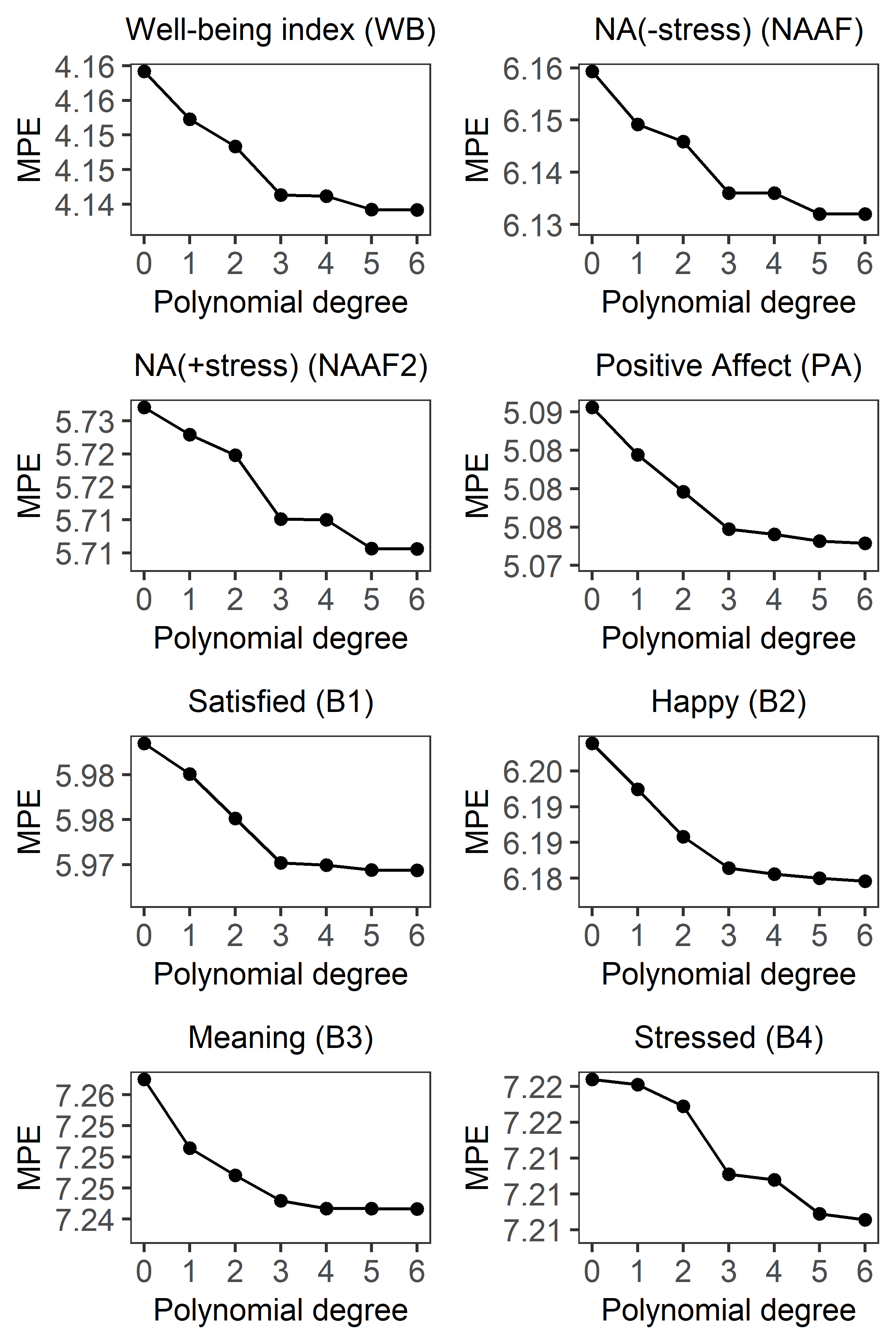

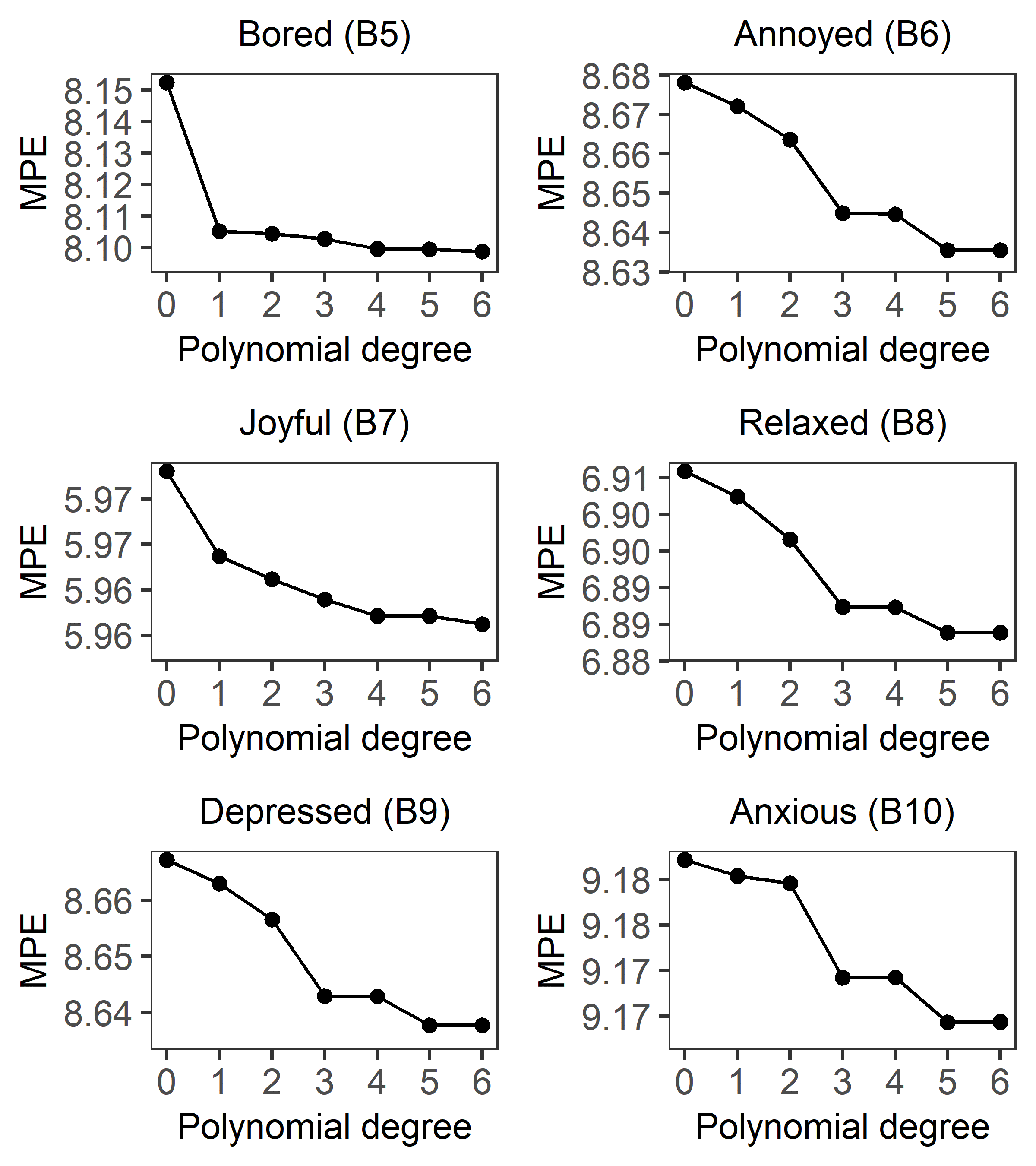


**S1 Fig.** *Results of the 10-fold Cross-Validation used to Choose the Optimal Degree of Polynomials for Each Measure*
