# Supplementary material for: How COVID-19 affected mental well-being: An 11- week trajectories of daily well-being of Koreans amidst COVID-19 by age, gender and region
Source: PLoS One. 2021 Apr 23;16(4):e0250252. doi: 10.1371/journal.pone.0250252 (PMC8064534; doi:10.1371/journal.pone.0250252)
Supplement: S2 Fig — (DOCX) [file pone.0250252.s002.docx]

**S2 Fig.** *Results of the Ten-fold Cross-Validation for the Well-being Index and the Well-being trajectory for Subsamples Providing two or more Daily Well-being Responses*


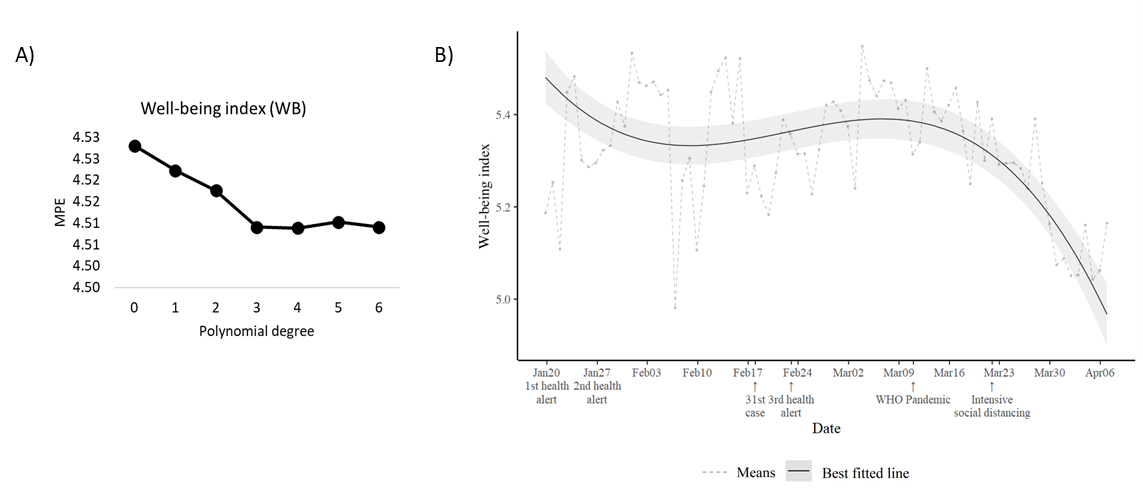


*Note.* We examined only those who provide daily well-being responses more than twice, excluding those who respond multiple times on the same day only (N = 28,060). Therefore, the number of participants and observations included in the analysis is 63,257 and 165,615, respectively. Panel A indicates the results of ten-fold cross-validation, showing that the cubic pattern (decline-recovery-decline) is the optimal degree of a polynomial model for the well-being index of the subsamples. Panel B is the estimated well-being trajectory corresponding to the result of cross-validation.
