# Supplementary material for: How COVID-19 affected mental well-being: An 11- week trajectories of daily well-being of Koreans amidst COVID-19 by age, gender and region
Source: PLoS One. 2021 Apr 23;16(4):e0250252. doi: 10.1371/journal.pone.0250252 (PMC8064534; doi:10.1371/journal.pone.0250252)
Supplement: S1 Table — (DOCX) [file pone.0250252.s003.docx]

| **S1 Table.**  *Questionnaire Items for the Well-being Index* |  |  |  |  |  |  |  |  |  |  |  |
| --- | --- | --- | --- | --- | --- | --- | --- | --- | --- | --- | --- |
| Instruction: Please respond to a momentary feeling. |  |  |  |  |  |  |  |  |  |  |  |
|  |  | | | | | | | | | | |
| Life satisfaction | Not at all----------------------------Very | | | | | | | | | | |
| 1. How satisfied are you with your life right now? | 0 | 1 | 2 | 3 | 4 | 5 | 6 | 7 | 8 | 9 | 10 |
|  |  |  |  |  |  |  |  |  |  |  |  |
| Positive affect |  |  |  |  |  |  |  |  |  |  |  |
| 2. How much are you feeling each emotion right now? |  |  |  |  |  |  |  |  |  |  |  |
| (1)   Happy | 0 | 1 | 2 | 3 | 4 | 5 | 6 | 7 | 8 | 9 | 10 |
| (2)   Joyful | 0 | 1 | 2 | 3 | 4 | 5 | 6 | 7 | 8 | 9 | 10 |
| (3)   Relaxed | 0 | 1 | 2 | 3 | 4 | 5 | 6 | 7 | 8 | 9 | 10 |
|  |  |  |  |  |  |  |  |  |  |  |  |
| Negative affect |  |  |  |  |  |  |  |  |  |  |  |
| 3. How much are you feeling each emotion right now? |  |  |  |  |  |  |  |  |  |  |  |
| (4)   Bored | 0 | 1 | 2 | 3 | 4 | 5 | 6 | 7 | 8 | 9 | 10 |
| (5)   Annoyed | 0 | 1 | 2 | 3 | 4 | 5 | 6 | 7 | 8 | 9 | 10 |
| (6)   Depressed | 0 | 1 | 2 | 3 | 4 | 5 | 6 | 7 | 8 | 9 | 10 |
| (7)   Anxious | 0 | 1 | 2 | 3 | 4 | 5 | 6 | 7 | 8 | 9 | 10 |
|  |  |  |  |  |  |  |  |  |  |  |  |
| Life meaning |  |  |  |  |  |  |  |  |  |  |  |
| 4. How meaningful do you feel your life is right now? | 0 | 1 | 2 | 3 | 4 | 5 | 6 | 7 | 8 | 9 | 10 |
|  |  |  |  |  |  |  |  |  |  |  |  |
| Stress |  |  |  |  |  |  |  |  |  |  |  |
| 5. How stressed are you right now? | 0 | 1 | 2 | 3 | 4 | 5 | 6 | 7 | 8 | 9 | 10 |
