# Supplementary material for: How COVID-19 affected mental well-being: An 11- week trajectories of daily well-being of Koreans amidst COVID-19 by age, gender and region
Source: PLoS One. 2021 Apr 23;16(4):e0250252. doi: 10.1371/journal.pone.0250252 (PMC8064534; doi:10.1371/journal.pone.0250252)
Supplement: S2 Table — (DOCX) [file pone.0250252.s004.docx]

| **S2 Table.**  *Results for the Multilevel Analyses on the Well-being Variables including Well-being Index, Positive Affect (PA), Negative Affect (NA), Life Satisfaction, and Life Meaning* | | | | |
| --- | --- | --- | --- | --- |
| Predictor | *Coefficient* | *SE* | *t* | *p* |
| Well-being Index |  |  |  |  |
| Intercept | 5.352 | .011 | 501.399 | .000 |
| Region | -.056 | .012 | -4.642 | .000 |
| Gender | .316 | .008 | 39.369 | .000 |
| Age _middle_ | -.104 | .007 | -15.091 | .000 |
| Age _old_ | .333 | .012 | 28.115 | .000 |
| Day | -1.634 | .087 | -18.887 | .000 |
| Day^2^ | 4.770 | .211 | 22.609 | .000 |
| Day^3^ | -3.771 | .144 | -26.207 | .000 |
| Positive Affect (PA) |  |  |  |  |
| Intercept | 5.681 | .012 | 455.841 | .000 |
| Region | -.042 | .013 | -3.200 | .001 |
| Gender | .270 | .009 | 30.599 | .000 |
| Age _middle_ | -.012 | .008 | -1.588 | .112 |
| Age _old_ | .281 | .013 | 21.634 | .000 |
| Day | -1.455 | .102 | -14.236 | .000 |
| Day^2^ | 4.408 | .248 | 17.767 | .000 |
| Day^3^ | -3.568 | .169 | -21.139 | .000 |
| Negative Affect (NA) |  |  |  |  |
| Intercept | 5.352 | .013 | 404.913 | .000 |
| Region | .038 | .014 | 2.748 | .006 |
| Gender | -.295 | .009 | -31.708 | .000 |
| Age _middle_ | .275 | .008 | 34.372 | .000 |
| Age _old_ | -.360 | .014 | -26.336 | .000 |
| Day | 1.857 | .109 | 17.114 | .000 |
| Day^2^ | -5.501 | .263 | -20.889 | .000 |
| Day^3^ | 4.263 | .179 | 23.805 | .000 |
| Life Satisfaction |  |  |  |  |
| Intercept | 5.959 | .013 | 444.050 | .000 |
| Region | -.064 | .014 | -4.443 | .000 |
| Gender | .326 | .010 | 33.890 | .000 |
| Age _middle_ | -.092 | .008 | -11.061 | .000 |
| Age _old_ | .183 | .014 | 12.892 | .000 |
| Day | -1.296 | .110 | -11.804 | .000 |
| Day^2^ | 4.323 | .267 | 16.203 | .000 |
| Day^3^ | -3.566 | .182 | -19.639 | .000 |
| Life meaning |  |  |  |  |
| Intercept | 5.497 | .015 | 375.226 | .000 |
| Region | -.098 | .016 | -6.188 | .000 |
| Gender | .411 | .011 | 38.622 | .000 |
| Age _middle_ | .184 | .009 | 20.139 | .000 |
| Age _old_ | .518 | .016 | 33.018 | .000 |
| Day | -1.276 | .120 | -10.674 | .000 |
| Day^2^ | 3.802 | .291 | 13.073 | .000 |
| Day^3^ | -3.156 | .198 | -15.934 | .000 |
| *Note.* Day was rescaled to the maximum value of 1. Each age group represented in the age variable was coded 1 and the other two groups were 0 (e.g., Age _middle_ = 1, Age _young_ and Age _old_ = 0). Region and Gender were dummy coded (Daegu-Gyeongbuk = 1, Other regions =0; Male = 1, Female = 0). | | | | |
