# Supplementary material for: How COVID-19 affected mental well-being: An 11- week trajectories of daily well-being of Koreans amidst COVID-19 by age, gender and region
Source: PLoS One. 2021 Apr 23;16(4):e0250252. doi: 10.1371/journal.pone.0250252 (PMC8064534; doi:10.1371/journal.pone.0250252)
Supplement: S3 Table — (DOCX) [file pone.0250252.s005.docx]

| **S3 Table.** | | | | |
| --- | --- | --- | --- | --- |
| *Results for the Multilevel Analyses on the Positive and Negative Emotion Variables* | | | | |
| Predictor | *Coefficient* | *SE* | *t* | *p* |
| Bored |  |  |  |  |
| Intercept | 5.373 | .009 | 566.723 | .000 |
| Region | .155 | .017 | 9.393 | .000 |
| Gender | -.210 | .011 | -18.959 | .000 |
| Age _middle_ | -.126 | .010 | -13.221 | .000 |
| Age _old_ | -.729 | .016 | -44.784 | .000 |
| Day | .668 | .013 | 50.540 | .000 |
| Annoyed |  |  |  |  |
| Intercept | 4.340 | .017 | 259.385 | .000 |
| Region | .056 | .017 | 3.259 | .001 |
| Gender | -.260 | .012 | -22.541 | .000 |
| Age _middle_ | .565 | .010 | 57.077 | .000 |
| Age _old_ | .040 | .017 | 2.346 | .019 |
| Day | 2.915 | .138 | 21.140 | .000 |
| Day^2^ | -8.750 | .334 | -26.196 | .000 |
| Day^3^ | 6.813 | .227 | 30.022 | .000 |
| Depressed |  |  |  |  |
| Intercept | 4.482 | .017 | 269.259 | .000 |
| Region | .033 | .017 | 1.899 | .058 |
| Gender | -.468 | .012 | -40.449 | .000 |
| Age _middle_ | .203 | .010 | 20.414 | .000 |
| Age _old_ | -.301 | .017 | -17.740 | .000 |
| Day | 2.356 | .137 | 17.200 | .000 |
| Day^2^ | -7.201 | .332 | -21.685 | .000 |
| Day^3^ | 5.636 | .226 | 24.971 | .000 |
| Anxious |  |  |  |  |
| Intercept | 4.646 | .017 | 271.682 | .000 |
| Region | .021 | .018 | 1.168 | .243 |
| Gender | -.414 | .012 | -34.721 | .000 |
| Age _middle_ | .132 | .010 | 12.820 | .000 |
| Age _old_ | -.414 | .018 | -23.583 | .000 |
| Day | 2.563 | .141 | 18.233 | .000 |
| Day^2^ | -6.684 | .341 | -19.605 | .000 |
| Day^3^ | 4.753 | .232 | 20.506 | .000 |
| Stress |  |  |  |  |
| Intercept | 5.965 | .015 | 391.360 | .000 |
| Region | .011 | .015 | 0.706 | .480 |
| Gender | -.251 | .010 | -24.075 | .000 |
| Age _middle_ | .361 | .009 | 40.313 | .000 |
| Age _old_ | -.368 | .015 | -24.107 | .000 |
| Day | 1.650 | .126 | 13.117 | .000 |
| Day^2^ | -5.318 | .305 | -17.464 | .000 |
| Day^3^ | 4.146 | .207 | 20.048 | .000 |
| Happy |  |  |  |  |
| Intercept | 5.906 | .014 | 428.868 | .000 |
| Region | -.053 | .015 | -3.601 | .000 |
| Gender | .284 | .010 | 28.927 | .000 |
| Age _middle_ | .012 | .008 | 1.365 | .172 |
| Age _old_ | .285 | .014 | 19.748 | .000 |
| Day | -1.161 | .113 | -10.288 | .000 |
| Day^2^ | 3.946 | .274 | 14.401 | .000 |
| Day^3^ | -3.377 | .186 | -18.111 | .000 |
| Joyful |  |  |  |  |
| Intercept | 5.398 | .008 | 664.234 | .000 |
| Region | -.048 | .014 | -3.420 | .001 |
| Gender | .328 | .010 | 34.544 | .000 |
| Age _middle_ | .015 | .008 | 1.836 | .066 |
| Age _old_ | .234 | .014 | 16.836 | .000 |
| Day | -.319 | .011 | -28.137 | .000 |
| Relaxed |  |  |  |  |
| Intercept | 5.721 | .015 | 381.121 | .000 |
| Region | -.025 | .015 | -1.657 | .098 |
| Gender | .197 | .010 | 19.138 | .000 |
| Age _middle_ | -.065 | .009 | -7.377 | .000 |
| Age _old_ | .334 | .015 | 22.072 | .000 |
| Day | -2.044 | .124 | -16.512 | .000 |
| Day^2^ | 6.228 | .300 | 20.773 | .000 |
| Day^3^ | -4.874 | .204 | -23.938 | .000 |
| *Note*. Day was rescaled to the maximum value of 1. Each age group represented in the age variable was coded 1 and the other two groups were 0 (e.g., Age _middle_ = 1, Age _young_ and Age _old_ = 0). Region and Gender were dummy coded (Daegu-Gyeongbuk = 1, Other regions =0; Male = 1, Female = 0). | | | | |
