# Supplementary material for: How COVID-19 affected mental well-being: An 11- week trajectories of daily well-being of Koreans amidst COVID-19 by age, gender and region
Source: PLoS One. 2021 Apr 23;16(4):e0250252. doi: 10.1371/journal.pone.0250252 (PMC8064534; doi:10.1371/journal.pone.0250252)
Supplement: S4 Table — (DOCX) [file pone.0250252.s006.docx]

| **S4 Table.** | | | | | |  |
| --- | --- | --- | --- | --- | --- | --- |
| *The Results for Model Comparison between a Baseline Model and a Day by Age Interaction Model for Each Well-being Measure* | | | | | |  |
| Model | AIC | BIC | Log likelihood | $\chi^{2}$(*df*) | *p* | |
| Well-being index |  |  |  |  |  | |
| Cubic model | 1963581 | 1963692 | -981781 |  |  | |
| Interaction model | 1963398 | 1963576 | -981683 | 195.35 (6) | <.001 | |
| Positive affect (PA) |  |  |  |  |  | |
| Cubic model | 2098814 | 2098925 | -1049397 |  |  | |
| Interaction model | 2098634 | 2098812 | -1049301 | 191.26 (6) | <.001 | |
| Negative affect (NA) |  |  |  |  |  | |
| Cubic model | 2154839 | 2154950 | -1077409 |  |  | |
| Interaction model | 2154748 | 2154925 | -1077358 | 103.17 (6) | <.001 | |
| Life satisfaction |  |  |  |  |  | |
| Cubic model | 2174099 | 2174210 | -1087039 |  |  | |
| Interaction model | 2173847 | 2174024 | -1086907 | 264.32 (6) | <.001 | |
| Life meaning |  |  |  |  |  | |
| Cubic model | 2263573 | 2263684 | -1131776 |  |  | |
| Interaction model | 2263225 | 2263402 | -1131596 | 360.18 (6) | <.001 | |
| Bored |  |  |  |  |  | |
| Linear model | 2357367 | 2357455 | -1178675 |  |  | |
| Interaction model | 2357173 | 2357284 | -1178576 | 197.75 (2) | <.001 | |
| Annoyed |  |  |  |  |  | |
| Cubic model | 2381797 | 2381908 | -1190888 |  |  | |
| Interaction model | 2381695 | 2381872 | -1190831 | 114.25 (6) | <.001 | |
| Depressed |  |  |  |  |  | |
| Cubic model | 2378206 | 2378317 | -1189093 |  |  | |
| Interaction model | 2378159 | 2378336 | -1189063 | 58.722 (6) | <.001 | |
| Anxious |  |  |  |  |  | |
| Cubic model | 2405485 | 2405596 | -1202733 |  |  | |
| Interaction model | 2405427 | 2405605 | -1202698 | 69.713 (6) | <.001 | |
| Stress |  |  |  |  |  | |
| Cubic model | 2289050 | 2289161 | -1144515 |  |  | |
| Interaction model | 2288973 | 2289151 | -1144471 | 89.235 (6) | <.001 | |
| Happy |  |  |  |  |  | |
| Cubic model | 2197599 | 2197710 | -1098790 |  |  | |
| Interaction model | 2197343 | 2197521 | -1098655 | 268.42 (6) | <.001 | |
| Joyful |  |  |  |  |  | |
| Linear model | 2207245 | 2207334 | -1103615 |  |  | |
| Interaction model | 2207113 | 2207224 | -1103547 | 135.65 (2) | <.001 | |
| Relaxed |  |  |  |  |  | |
| Cubic model | 2274761 | 2274872 | -1137371 |  |  | |
| Interaction model | 2274666 | 2274844 | -1137317 | 107.08 (6) | <.001 | |
|  | | | | | |  |
