# Supplementary material for: How COVID-19 affected mental well-being: An 11- week trajectories of daily well-being of Koreans amidst COVID-19 by age, gender and region
Source: PLoS One. 2021 Apr 23;16(4):e0250252. doi: 10.1371/journal.pone.0250252 (PMC8064534; doi:10.1371/journal.pone.0250252)
Supplement: S5 Table — (DOCX) [file pone.0250252.s007.docx]

| **S5 Table.**  *Results for Examining Day by Age Interaction on Negative Emotion Measures* | | | | |  |
| --- | --- | --- | --- | --- | --- |
| Predictor | Coefficient | *SE* | *t* | *p* | |
| Bored |  |  |  |  | |
| Intercept | 5.289 | .011 | 464.959 | .000 | |
| Region | .154 | .017 | 9.358 | .000 | |
| Gender | -.210 | .011 | -18.912 | .000 | |
| Age _middle_ | .026 | .017 | 1.525 | .127 | |
| Age _old_ | -.470 | .028 | -16.869 | .000 | |
| Day | .833 | .018 | 45.935 | .000 | |
| Day x Age _middle_ | -.306 | .028 | -10.862 | .000 | |
| Day x Age _old_ | -.529 | .047 | -11.371 | .000 | |
| Annoyed |  |  |  |  | |
| Intercept | 4.352 | .022 | 197.818 | .000 | |
| Region | .056 | .017 | 3.281 | .001 | |
| Gender | -.262 | .012 | -22.718 | .000 | |
| Age _middle_ | .550 | .034 | 16.205 | .000 | |
| Age _old_ | .031 | .056 | .547 | .584 | |
| Day | 3.048 | .189 | 16.133 | .000 | |
| Day^2^ | -9.652 | .457 | -21.137 | .000 | |
| Day^3^ | 7.692 | .309 | 24.893 | .000 | |
| Day x Age _middle_ | -.233 | .294 | -.794 | .427 | |
| Day x Age _old_ | -1.036 | .498 | -2.082 | .037 | |
| Day^2^ x Age _middle_ | 1.539 | .712 | 2.162 | .031 | |
| Day^2^ x Age _old_ | 4.859 | 1.210 | 4.015 | .000 | |
| Day^3^ x Age _middle_ | -1.508 | .485 | -3.112 | .002 | |
| Day^3^ x Age _old_ | -4.377 | .824 | -5.313 | .000 | |
| Depressed |  |  |  |  | |
| Intercept | 4.454 | .022 | 203.651 | .000 | |
| Region | .033 | .017 | 1.913 | .056 | |
| Gender | -.469 | .012 | -40.529 | .000 | |
| Age _middle_ | .254 | .034 | 7.517 | .000 | |
| Age _old_ | -.175 | .056 | -3.150 | .002 | |
| Day | 2.681 | .188 | 14.278 | .000 | |
| Day^2^ | -8.194 | .454 | -18.041 | .000 | |
| Day^3^ | 6.413 | .307 | 20.860 | .000 | |
| Day x Age _middle_ | -.539 | .292 | -1.846 | .065 | |
| Day x Age _old_ | -1.701 | .494 | -3.446 | .001 | |
| Day^2^ x Age _middle_ | 1.565 | .708 | 2.211 | .027 | |
| Day^2^ x Age _old_ | 5.360 | 1.201 | 4.464 | .000 | |
| Day^3^ x Age _middle_ | -1.203 | .482 | -2.496 | .013 | |
| Day^3^ x Age _old_ | -4.257 | .818 | -5.207 | .000 | |
| Anxious |  |  |  |  | |
| Intercept | 4.649 | .022 | 207.039 | .000 | |
| Region | .021 | .018 | 1.190 | .234 | |
| Gender | -.416 | .012 | -34.854 | .000 | |
| Age _middle_ | 2.764 | .193 | 14.342 | .000 | |
| Age _old_ | .132 | .035 | 3.815 | .000 | |
| Day | -.415 | .057 | -7.273 | .000 | |
| Day^2^ | -7.625 | .466 | -16.349 | .000 | |
| Day^3^ | 5.581 | .316 | 17.671 | .000 | |
| Day x Age _middle_ | -.334 | .299 | -1.115 | .265 | |
| Day x Age _old_ | -1.175 | .506 | -2.323 | .020 | |
| Day^2^ x Age _middle_ | 1.495 | .727 | 2.056 | .040 | |
| Day^2^ x Age _old_ | 4.911 | 1.231 | 3.990 | .000 | |
| Day^3^ x Age _middle_ | -1.314 | .495 | -2.654 | .008 | |
| Day^3^ x Age _old_ | -4.194 | .838 | -5.003 | .000 | |
| Stress |  |  |  |  | |
| Intercept | 5.960 | .020 | 297.209 | .000 | |
| Region | .011 | .015 | .722 | .470 | |
| Gender | -.252 | .010 | -24.175 | .000 | |
| Age _middle_ | .346 | .031 | 11.162 | .000 | |
| Age _old_ | -.212 | .051 | -4.125 | .000 | |
| Day | 1.752 | .172 | 10.168 | .000 | |
| Day^2^ | -5.864 | .416 | -14.091 | .000 | |
| Day^3^ | 4.683 | .281 | 16.640 | .000 | |
| Day x Age _middle_ | .140 | .268 | .525 | .600 | |
| Day x Age _old_ | -2.181 | .455 | -4.793 | .000 | |
| Day^2^ x Age _middle_ | .172 | .649 | .265 | .791 | |
| Day^2^ x Age _old_ | 6.558 | 1.106 | 5.929 | .000 | |
| Day^3^ x Age _middle_ | -.444 | .442 | -1.006 | .314 | |
| Day^3^ x Age _old_ | 5.011 | .753 | -6.658 | .000 | |
| *Note.* Day was rescaled to the maximum value of 1. Each age group represented in the age variable was coded 1 and the other two groups were 0 (e.g., Age _middle_ = 1, Age _young_ and Age _old_ = 0). Region and Gender were dummy coded (Daegu-Gyeongbuk = 1, Other regions =0; Male = 1, Female = 0). | | | | |  |
