# Supplementary material for: How COVID-19 affected mental well-being: An 11- week trajectories of daily well-being of Koreans amidst COVID-19 by age, gender and region
Source: PLoS One. 2021 Apr 23;16(4):e0250252. doi: 10.1371/journal.pone.0250252 (PMC8064534; doi:10.1371/journal.pone.0250252)
Supplement: S6 Table — (DOCX) [file pone.0250252.s008.docx]

| **S6 Table.**  *Results for Examining Day by Age Interaction on Positive Emotion Measures* | | | | |
| --- | --- | --- | --- | --- |
| Predictor | Coefficient | *SE* | *t* | *p* |
| Happy |  |  |  |  |
| Intercept | 5.938 | .018 | 328.953 | .000 |
| Region | -.052 | .015 | -3.564 | .000 |
| Gender | .283 | .010 | 28.896 | .000 |
| Age _middle_ | -.071 | .028 | -2.540 | .011 |
| Age _old_ | .252 | .046 | 5.514 | .000 |
| Day | -.870 | .155 | -5.621 | .000 |
| Day^2^ | 2.967 | .375 | 7.911 | .000 |
| Day^3^ | -2.781 | .254 | -10.937 | .000 |
| Day x Age _middle_ | -.453 | .240 | -1.885 | .059 |
| Day x Age _old_ | -.592 | .404 | -1.466 | .143 |
| Day^2^ x Age _middle_ | 1.837 | .584 | 3.144 | .002 |
| Day^2^ x Age _old_ | 1.065 | .984 | 1.082 | .279 |
| Day^3^ x Age _middle_ | -1.201 | .398 | -3.014 | .003 |
| Day^3^ x Age _old_ | -.077 | .671 | -0.115 | .909 |
| Joyful |  |  |  |  |
| Intercept | 5.459 | .010 | 559.616 | .000 |
| Region | -.048 | .014 | -3.388 | .001 |
| Gender | .328 | .010 | 34.506 | .000 |
| Age _middle_ | -.103 | .014 | -7.133 | .000 |
| Age _old_ | .068 | .024 | 2.853 | .004 |
| Day | -.441 | .016 | -28.310 | .000 |
| Day x Age _middle_ | .239 | .024 | 9.856 | .000 |
| Day x Age _old_ | .339 | .040 | 8.476 | .000 |
| Relaxed |  |  |  |  |
| Intercept | 5.727 | .020 | 290.086 | .000 |
| Region | -.025 | .015 | -1.656 | .098 |
| Gender | .198 | .010 | 19.234 | .000 |
| Age _middle_ | -.098 | .030 | -3.204 | .001 |
| Age _old_ | .367 | .050 | 7.274 | .000 |
| Day | -1.996 | .170 | -11.766 | .000 |
| Day^2^ | 6.315 | .410 | 15.412 | .000 |
| Day^3^ | -5.104 | .277 | -18.413 | .000 |
| Day x Age _middle_ | -.008 | .263 | -0.031 | .975 |
| Day x Age _old_ | .070 | .447 | 0.158 | .875 |
| Day^2^ x Age _middle_ | -.174 | .639 | -0.272 | .786 |
| Day^2^ x Age _old_ | -1.729 | 1.088 | -1.590 | .112 |
| Day^3^ x Age _middle_ | .374 | .435 | 0.860 | .390 |
| Day^3^ x Age _old_ | 2.044 | .740 | 2.761 | .006 |
| *Note.* Day was rescaled to the maximum value of 1. Each age group represented in the age variable was coded 1 and the other two groups were 0 (e.g., Age _middle_ = 1, Age _young_ and Age _old_ = 0). Region and Gender were dummy coded (Daegu-Gyeongbuk = 1, Other regions =0; Male = 1, Female = 0). | | | | |
