# Supplementary material for: How COVID-19 affected mental well-being: An 11- week trajectories of daily well-being of Koreans amidst COVID-19 by age, gender and region
Source: PLoS One. 2021 Apr 23;16(4):e0250252. doi: 10.1371/journal.pone.0250252 (PMC8064534; doi:10.1371/journal.pone.0250252)
Supplement: S7 Table — (DOCX) [file pone.0250252.s009.docx]

| **S7 Table.** | | | | |
| --- | --- | --- | --- | --- |
| *Results for Multilevel Analyses Examining the Effects of Individual Characteristics on All Well-being Measures* | | | | |
| Predictor | *Coefficient* | *SE* | *t* | *p* |
| Well-being Index |  |  |  |  |
| Intercept | 5.172 | .005 | 1052.539 | .000 |
| Region | -.059 | .012 | -4.915 | .000 |
| Gender | .310 | .008 | 38.535 | .000 |
| Age _middle_ | -.097 | .007 | -13.988 | .000 |
| Age _old_ | .352 | .012 | 29.599 | .000 |
| Positive Affect |  |  |  |  |
| Intercept | 5.521 | .005 | 1023.855 | .000 |
| Region | -.045 | .013 | -3.449 | .001 |
| Gender | .265 | .009 | 29.901 | .000 |
| Age _middle_ | -.005 | .008 | -0.601 | .548 |
| Age _old_ | .299 | .013 | 22.998 | .000 |
| Negative Affect | |  |  |  |
| Intercept | 5.523 | .006 | 973.178 | .000 |
| Region | .040 | .014 | 2.918 | .004 |
| Gender | -.289 | .009 | -30.989 | .000 |
| Age _middle_ | .269 | .008 | 33.638 | .000 |
| Age _old_ | -.377 | .014 | -27.553 | .000 |
| Life satisfaction |  |  |  |  |
| Intercept | 5.850 | .006 | 995.837 | .000 |
| Region | -.066 | .014 | -4.597 | .000 |
| Gender | .321 | .010 | 33.299 | .000 |
| Age _middle_ | -.086 | .008 | -10.337 | .000 |
| Age _old_ | .200 | .014 | 14.071 | .000 |
| Life meaning |  |  |  |  |
| Intercept | 5.327 | .007 | 819.269 | .000 |
| Region | -.102 | .016 | -6.444 | .000 |
| Gender | .406 | .011 | 38.053 | .000 |
| Age _middle_ | .193 | .009 | 21.107 | .000 |
| Age _old_ | .537 | .016 | 34.196 | .000 |
| Bored |  |  |  |  |
| Intercept | 5.710 | .007 | 843.694 | .000 |
| Region | .165 | .017 | 9.932 | .000 |
| Gender | -.207 | .011 | -18.584 | .000 |
| Age _middle_ | -.142 | .010 | -14.860 | .000 |
| Age _old_ | -.745 | .016 | -45.656 | .000 |
| Annoyed |  |  |  |  |
| Intercept | 4.600 | .007 | 654.569 | .000 |
| Region | .060 | .017 | 3.463 | .001 |
| Gender | -.250 | .012 | -21.629 | .000 |
| Age _middle_ | .557 | .010 | 56.117 | .000 |
| Age _old_ | .013 | .017 | 0.756 | .450 |
| Depressed | |  |  |  |
| Intercept | 4.683 | .007 | 664.560 | .000 |
| Region | .035 | .017 | 2.057 | .040 |
| Gender | -.459 | .012 | -39.691 | .000 |
| Age _middle_ | .196 | .010 | 19.707 | .000 |
| Age _old_ | -.324 | .017 | -19.029 | .000 |
| Anxious |  |  |  |  |
| Intercept | 4.896 | .007 | 673.266 | .000 |
| Region | .023 | .018 | 1.277 | .202 |
| Gender | -.408 | .012 | -34.139 | .000 |
| Age _middle_ | .128 | .010 | 12.487 | .000 |
| Age _old_ | -.427 | .018 | -24.352 | .000 |
| Stress |  |  |  |  |
| Intercept | 6.063 | .006 | 956.951 | .000 |
| Region | .011 | .016 | 0.740 | .459 |
| Gender | -.245 | .010 | -23.534 | .000 |
| Age _middle_ | .358 | .009 | 40.026 | .000 |
| Age _old_ | -.382 | .015 | -24.984 | .000 |
| Happy |  |  |  |  |
| Intercept | 5.786 | .006 | 967.134 | .000 |
| Region | -.056 | .015 | -3.820 | .000 |
| Gender | .278 | .010 | 28.328 | .000 |
| Age _middle_ | .019 | .008 | 2.302 | .021 |
| Age _old_ | .304 | .014 | 21.043 | .000 |
| Joyful |  |  |  |  |
| Intercept | 5.237 | .006 | 906.273 | .000 |
| Region | -.053 | .014 | -3.735 | .000 |
| Gender | .327 | .010 | 34.337 | .000 |
| Age _middle_ | .023 | .008 | 2.766 | .006 |
| Age _old_ | .242 | .014 | 17.384 | .000 |
| Relaxed |  |  |  |  |
| Intercept | 5.545 | .006 | 884.140 | .000 |
| Region | -.028 | .015 | -1.817 | .069 |
| Gender | .190 | .010 | 18.423 | .000 |
| Age _middle_ | -.059 | .009 | -6.689 | .000 |
| Age _old_ | .353 | .015 | 23.328 | .000 |
| *Note*. Day was rescaled to the maximum value of 1. Each age group represented in the age variable was coded 1 and the other two groups were 0 (e.g., Age _middle_ = 1, Age _young_ and Age _old_ = 0). Region and Gender were dummy coded (Daegu-Gyeongbuk = 1, Other regions =0; Male = 1, Female = 0). | | | | |
