# Supplementary material for: How COVID-19 affected mental well-being: An 11- week trajectories of daily well-being of Koreans amidst COVID-19 by age, gender and region
Source: PLoS One. 2021 Apr 23;16(4):e0250252. doi: 10.1371/journal.pone.0250252 (PMC8064534; doi:10.1371/journal.pone.0250252)
Supplement: S8 Table — (DOCX) [file pone.0250252.s010.docx]

| **S8 Table.** | | | | | |  |
| --- | --- | --- | --- | --- | --- | --- |
| *The Results for Model Comparison between a Baseline Model and a Day by Region Interaction Model for Each Well-being Measure* | | | | | |  |
| Model | AIC | BIC | Log likelihood | $\chi^{2}$(df) | *p* | |
| Well-being index |  |  |  |  |  | |
| Cubic model | 1963581 | 1963692 | -981781 |  |  | |
| Interaction model | 1963580 | 1963724 | -981777 | 7.318 (3) | .062 | |
| Positive affect (PA) |  |  |  |  |  | |
| Cubic model | 2098814 | 2098925 | -1049397 |  |  | |
| Interaction model | 2098817 | 2098962 | -1049396 | 2.303 (3) | .512 | |
| Negative affect (NA) |  |  |  |  |  | |
| Cubic model | 2154839 | 2154950 | -1077409 |  |  | |
| Interaction model | 2154836 | 2154980 | -1077405 | 9.120 (3) | .028 | |
| Life satisfaction |  |  |  |  |  | |
| Cubic model | 2174099 | 2174210 | -1087039 |  |  | |
| Interaction model | 2174103 | 2174248 | -1087039 | 1.535 (3) | .674 | |
| Life meaning |  |  |  |  |  | |
| Cubic model | 2263573 | 2263684 | -1131776 |  |  | |
| Interaction model | 2263576 | 2263721 | -1131775 | 2.563 (3) | .464 | |
| Bored |  |  |  |  |  | |
| Linear model | 2357367 | 2357455 | -1178675 |  |  | |
| Interaction model | 2357357 | 2357457 | -1178670 | 11.464 (1) | .001 | |
| Annoyed |  |  |  |  |  | |
| Cubic model | 2381797 | 2381908 | -1190888 |  |  | |
| Interaction model | 2381798 | 2381943 | -1190886 | 4.666 (3) | .198 | |
| Depressed |  |  |  |  |  | |
| Cubic model | 2378206 | 2378317 | -1189093 |  |  | |
| Interaction model | 2378210 | 2378354 | -1189092 | 1.657 (3) | .647 | |
| Anxious |  |  |  |  |  | |
| Cubic model | 2405485 | 2405596 | -1202733 |  |  | |
| Interaction model | 2405487 | 2405631 | -1202731 | 3.973 (3) | .264 | |
| Stress |  |  |  |  |  | |
| Cubic model | 2289050 | 2289161 | -1144515 |  |  | |
| Interaction model | 2289045 | 2289189 | -1144510 | 11.182 (3) | .011 | |
| Happy |  |  |  |  |  | |
| Cubic model | 2197599 | 2197710 | -1098790 |  |  | |
| Interaction model | 2197604 | 2197748 | -1098789 | 1.752 (3) | .625 | |
| Joyful |  |  |  |  |  | |
| Linear model | 2207245 | 2207334 | -1103615 |  |  | |
| Interaction model | 2207245 | 2207345 | -1103614 | 1.864 (1) | .172 | |
| Relaxed |  |  |  |  |  | |
| Cubic model | 2274761 | 2274872 | -1137371 |  |  | |
| Interaction model | 2274763 | 2274908 | -1137369 | 4.117 (3) | .249 | |
| *Note.* Day was rescaled to have a range from 0 to 1. Each age group represented in the age variable was coded 1 and the other two groups were 0 (e.g., Age _middle_ = 1, Age _young_ and Age _old_ = 0). Region and Gender were dummy coded (Daegu-Gyeongbuk = 1, Other regions =0; Male = 1, Female = 0). | | | | | |  |
