# Supplementary material for: How COVID-19 affected mental well-being: An 11- week trajectories of daily well-being of Koreans amidst COVID-19 by age, gender and region
Source: PLoS One. 2021 Apr 23;16(4):e0250252. doi: 10.1371/journal.pone.0250252 (PMC8064534; doi:10.1371/journal.pone.0250252)
Supplement: S9 Table — (DOCX) [file pone.0250252.s011.docx]

| **S9 Table.** | | | | |
| --- | --- | --- | --- | --- |
| *Results for Examining Day by Region Interaction on Well-being Measures including Well-being Index, Positive Affect (PA), Negative Affect (NA), Life Satisfaction, and Life meaning* | | | | |
| Predictor | Coefficient | *SE* | *t* | *p* |
| Well-being index |  |  |  |  |
| Intercept | 5.346 | .011 | 485.044 | .000 |
| Region | .026 | .038 | .689 | .491 |
| Gender | .316 | .008 | 39.368 | .000 |
| Age _middle_ | -.104 | .007 | -15.094 | .000 |
| Age _old_ | .333 | .012 | 28.113 | .000 |
| Day | -1.597 | .090 | -17.713 | .000 |
| Day^2^ | 4.702 | .220 | 21.377 | .000 |
| Day^3^ | -3.731 | .150 | -24.855 | .000 |
| Day x Region | -.488 | .322 | -1.518 | .129 |
| Day^2^ x Region | .878 | .778 | 1.128 | .259 |
| Day^3^ x Region | -.516 | .528 | -.977 | .329 |
| Positive affect (PA) |  |  |  |  |
| Intercept | 5.676 | .013 | 440.797 | .000 |
| Region | .015 | .044 | .342 | .732 |
| Gender | .270 | .009 | 30.598 | .000 |
| Age _middle_ | -.012 | .008 | -1.590 | .112 |
| Age _old_ | .281 | .013 | 21.634 | .000 |
| Day | -1.427 | .106 | -13.403 | .000 |
| Day^2^ | 4.355 | .259 | 16.838 | .000 |
| Day^3^ | -3.536 | .176 | -20.085 | .000 |
| Day x Region | -.367 | .380 | -.966 | .334 |
| Day^2^ x Region | .687 | .915 | .750 | .453 |
| Day^3^ x Region | -.407 | .619 | -.657 | .511 |
| Negative affect (NA) |  |  |  |  |
| Intercept | 5.359 | .014 | 392.373 | .000 |
| Region | -.061 | .047 | -1.287 | .198 |
| Gender | -.295 | .009 | -31.707 | .000 |
| Age _middle_ | .275 | .008 | 34.375 | .000 |
| Age _old_ | -.360 | .014 | -26.337 | .000 |
| Day | 1.806 | .113 | 15.974 | .000 |
| Day^2^ | -5.387 | .275 | -19.624 | .000 |
| Day^3^ | 4.184 | .187 | 22.401 | .000 |
| Day x Region | .659 | .404 | 1.632 | .103 |
| Day^2^ x Region | -1.427 | .972 | -1.469 | .142 |
| Day^3^ x Region | .969 | .657 | 1.476 | .140 |
| Life satisfaction |  |  |  |  |
| Intercept | 5.958 | .014 | 429.777 | .000 |
| Region | -.062 | .048 | -1.306 | .192 |
| Gender | .326 | .010 | 33.890 | .000 |
| Age _middle_ | -.092 | .008 | -11.061 | .000 |
| Age _old_ | .183 | .014 | 12.892 | .000 |
| Day | -1.297 | .114 | -11.342 | .000 |
| Day^2^ | 4.316 | .278 | 15.517 | .000 |
| Day^3^ | -3.554 | .189 | -18.761 | .000 |
| Day x Region | .024 | .409 | .060 | .952 |
| Day^2^ x Region | .061 | .984 | .062 | .950 |
| Day^3^ x Region | -.133 | .666 | -.199 | .842 |
| Life meaning |  |  |  |  |
| Intercept | 5.491 | .015 | 362.854 | .000 |
| Region | -.023 | .052 | -.441 | .659 |
| Gender | .411 | .011 | 38.621 | .000 |
| Age _middle_ | .184 | .009 | 20.138 | .000 |
| Age _old_ | .518 | .016 | 33.019 | .000 |
| Day | -1.234 | .125 | -9.909 | .000 |
| Day^2^ | 3.716 | .303 | 12.257 | .000 |
| Day^3^ | -3.103 | .207 | -15.018 | .000 |
| Day x Region | -.545 | .445 | -1.225 | .221 |
| Day^2^ x Region | 1.102 | 1.073 | 1.027 | .304 |
| Day^3^ x Region | -.675 | .726 | -.929 | .353 |
| *Note.* Day was rescaled to the maximum value of 1. Each age group represented in the age variable was coded 1 and the other two groups were 0 (e.g., Age _middle_ = 1, Age _young_ and Age _old_ = 0). Region and Gender were dummy coded (Daegu = 1, Other regions =0; Male = 1, Female = 0). | | | | |
