# Supplementary material for: How COVID-19 affected mental well-being: An 11- week trajectories of daily well-being of Koreans amidst COVID-19 by age, gender and region
Source: PLoS One. 2021 Apr 23;16(4):e0250252. doi: 10.1371/journal.pone.0250252 (PMC8064534; doi:10.1371/journal.pone.0250252)
Supplement: S10 Table — (DOCX) [file pone.0250252.s012.docx]

| **S10 Table.** | | | | |
| --- | --- | --- | --- | --- |
| *Results for Examining Day by Region Interaction on Negative Emotion Measures* | | | | |
| Predictor | Coefficient | *SE* | *t* | *p* |
| Bored |  |  |  |  |
| Intercept | 5.379 | .010 | 556.090 | 0.000 |
| Region | .072 | .030 | 2.425 | 0.015 |
| Gender | -.210 | .011 | -18.958 | 0.000 |
| Age _middle_ | -.126 | .010 | -13.216 | 0.000 |
| Age _old_ | -.729 | .016 | -44.779 | 0.000 |
| Day | .654 | .014 | 47.498 | 0.000 |
| Day x Region | .164 | .048 | 3.386 | 0.001 |
| Annoyed |  |  |  |  |
| Intercept | 4.345 | .017 | 251.186 | 0.000 |
| Region | -.001 | .060 | -0.015 | 0.988 |
| Gender | -.260 | .012 | -22.540 | 0.000 |
| Age _middle_ | .565 | .010 | 57.078 | 0.000 |
| Age _old_ | .040 | .017 | 2.345 | 0.019 |
| Day | 2.885 | .144 | 20.092 | 0.000 |
| Day^2^ | -8.673 | .348 | -24.906 | 0.000 |
| Day^3^ | 6.751 | .237 | 28.521 | 0.000 |
| Day x Region | .367 | .514 | 0.715 | 0.474 |
| Day^2^ x Region | -.940 | 1.233 | -0.763 | 0.446 |
| Day^3^ x Region | .738 | .832 | 0.886 | 0.375 |
| Depressed |  |  |  |  |
| Intercept | 4.484 | .017 | 260.622 | 0.000 |
| Region | .008 | .060 | 0.132 | 0.895 |
| Gender | -.468 | .012 | -40.449 | 0.000 |
| Age _middle_ | .203 | .010 | 20.414 | 0.000 |
| Age _old_ | -.301 | .017 | -17.742 | 0.000 |
| Day | 2.335 | .143 | 16.368 | 0.000 |
| Day^2^ | -7.136 | .346 | -20.613 | 0.000 |
| Day^3^ | 5.583 | .235 | 23.715 | 0.000 |
| Day x Region | .254 | .510 | 0.497 | 0.619 |
| Day^2^ x Region | -.784 | 1.225 | -0.640 | 0.522 |
| Day^3^ x Region | .631 | .828 | 0.762 | 0.446 |
| Anxious |  |  |  |  |
| Intercept | 4.653 | .018 | 263.279 | 0.000 |
| Region | -.073 | .061 | -1.190 | 0.234 |
| Gender | -.414 | .012 | -34.721 | 0.000 |
| Age _middle_ | .132 | .010 | 12.819 | 0.000 |
| Age _old_ | -.414 | .018 | -23.588 | 0.000 |
| Day | 2.487 | .146 | 16.983 | 0.000 |
| Day^2^ | -6.492 | .355 | -18.267 | 0.000 |
| Day^3^ | 4.622 | .242 | 19.117 | 0.000 |
| Day x Region | .967 | .524 | 1.848 | 0.065 |
| Day^2^ x Region | -2.397 | 1.258 | -1.905 | 0.057 |
| Day^3^ x Region | 1.624 | .850 | 1.911 | 0.056 |
| Stress |  |  |  |  |
| Intercept | 5.972 | .016 | 378.988 | 0.000 |
| Region | -.073 | .055 | -1.343 | 0.179 |
| Gender | -.251 | .010 | -24.075 | 0.000 |
| Age _middle_ | .361 | .009 | 40.314 | 0.000 |
| Age _old_ | -.368 | .015 | -24.110 | 0.000 |
| Day | 1.597 | .131 | 12.193 | 0.000 |
| Day^2^ | -5.175 | .317 | -16.301 | 0.000 |
| Day^3^ | 4.034 | .216 | 18.703 | 0.000 |
| Day x Region | .653 | .469 | 1.394 | 0.163 |
| Day^2^ x Region | -1.740 | 1.124 | -1.548 | 0.122 |
| Day^3^ x Region | 1.336 | .758 | 1.762 | 0.078 |
| *Note.* Day was rescaled to the maximum value of 1. Each age group represented in the age variable was coded 1 and the other two groups were 0 (e.g., Age _middle_ = 1, Age _young_ and Age _old_ = 0). Region and Gender were dummy coded (Daegu = 1, Other regions =0; Male = 1, Female = 0). | | | | |
