# Supplementary material for: How COVID-19 affected mental well-being: An 11- week trajectories of daily well-being of Koreans amidst COVID-19 by age, gender and region
Source: PLoS One. 2021 Apr 23;16(4):e0250252. doi: 10.1371/journal.pone.0250252 (PMC8064534; doi:10.1371/journal.pone.0250252)
Supplement: S11 Table — (DOCX) [file pone.0250252.s013.docx]

| **S11 Table.** | | | | |
| --- | --- | --- | --- | --- |
| *Results for Examining Day by Region Interaction on Positive Emotion Measures* | | | | |
| Predictor | Coefficient | *SE* | *t* | *p* |
| Happy |  |  |  |  |
| Intercept | 5.907 | .014 | 415.136 | .000 |
| Region | -.068 | .049 | -1.395 | .163 |
| Gender | .284 | .010 | 28.927 | .000 |
| Age _middle_ | .012 | .008 | 1.365 | .172 |
| Age _old_ | .285 | .014 | 19.748 | .000 |
| Day | -1.174 | .118 | -9.992 | .000 |
| Day^2^ | 3.966 | .286 | 13.883 | .000 |
| Day^3^ | -3.381 | .194 | -17.384 | .000 |
| Day x Region | .183 | .420 | .436 | .663 |
| Day^2^ x Region | -.285 | 1.011 | -.282 | .778 |
| Day^3^ x Region | .079 | .684 | .116 | .907 |
| Joyful |  |  |  |  |
| Intercept | 5.395 | .008 | 650.665 | .000 |
| Region | -.020 | .025 | -.768 | .443 |
| Gender | .328 | .010 | 34.543 | .000 |
| Age _middle_ | .015 | .008 | 1.834 | .067 |
| Age _old_ | .234 | .014 | 16.834 | .000 |
| Day | -.315 | .012 | -26.590 | .000 |
| Day x Region | -.057 | .042 | -1.365 | .172 |
| Relaxed |  |  |  |  |
| Intercept | 5.714 | .016 | 368.185 | .000 |
| Region | .075 | .054 | 1.401 | .161 |
| Gender | .197 | .010 | 19.137 | .000 |
| Age _middle_ | -.065 | .009 | -7.377 | .000 |
| Age _old_ | .334 | .015 | 22.075 | .000 |
| Day | -1.976 | .129 | -15.329 | .000 |
| Day^2^ | 6.076 | .313 | 19.441 | .000 |
| Day^3^ | -4.777 | .212 | -22.489 | .000 |
| Day x Region | -.870 | .461 | -1.887 | .059 |
| Day^2^ x Region | 1.933 | 1.107 | 1.747 | .081 |
| Day^3^ x Region | -1.231 | .747 | -1.649 | .099 |
| *Note.* Day was rescaled to the maximum value of 1. Each age group represented in the age variable was coded 1 and the other two groups were 0 (e.g., Age _middle_ = 1, Age _young_ and Age _old_ = 0). Region and Gender were dummy coded (Daegu-Gyeongbuk = 1, Other regions =0; Male = 1, Female = 0). | | | | |
