# Supplementary material for: How COVID-19 affected mental well-being: An 11- week trajectories of daily well-being of Koreans amidst COVID-19 by age, gender and region
Source: PLoS One. 2021 Apr 23;16(4):e0250252. doi: 10.1371/journal.pone.0250252 (PMC8064534; doi:10.1371/journal.pone.0250252)
Supplement: S12 Table — (DOCX) [file pone.0250252.s014.docx]

| **S12 Table.**  *Results for Examining Day by Age Interaction on Various Well-being Measures including Well-being Index, Positive Affect (PA), Negative Affect (NA), Life Satisfaction, and Life Meaning* | | | | |
| --- | --- | --- | --- | --- |
| Predictor | Coefficient | *SE* | *t* | *p* |
| Well-being index |  |  |  |  |
| Intercept | 5.366 | .014 | 385.485 | .000 |
| Region | -.055 | .012 | -4.629 | .000 |
| Gender | .316 | .008 | 39.422 | .000 |
| Age _middle_ | -.134 | .021 | -6.235 | .000 |
| Age _old_ | .289 | .035 | 8.301 | .000 |
| Day | -1.528 | .119 | -12.855 | .000 |
| Day^2^ | 4.546 | .279 | 15.707 | .000 |
| Day^3^ | -3.735 | .197 | -18.978 | .000 |
| Day x Age _middle_ | -.235 | .185 | -1.271 | .204 |
| Day x Age _old_ | .286 | .306 | .936 | .349 |
| Day^2^ x Age _middle_ | .655 | .451 | 1.454 | .146 |
| Day^2^ x Age _old_ | -1.549 | .747 | -2.073 | .038 |
| Day^3^ x Age _middle_ | -.284 | .308 | -.922 | .357 |
| Day^3^ x Age _old_ | 1.686 | .510 | 3.305 | .001 |
| Positive affect |  |  |  |  |
| Intercept | 5.699 | .016 | 348.741 | .000 |
| Region | -.042 | .013 | -3.174 | .002 |
| Gender | .270 | .009 | 30.607 | .000 |
| Age _middle_ | -.066 | .025 | -2.613 | .009 |
| Age _old_ | .277 | .041 | 6.693 | .000 |
| Day | -1.246 | .140 | -8.891 | .000 |
| Day^2^ | 3.798 | .340 | 11.185 | .000 |
| Day^3^ | -3.244 | .230 | -14.096 | .000 |
| Day x Age _middle_ | -.309 | .218 | -1.420 | .156 |
| Day x Age _old_ | -.426 | .366 | -1.164 | .244 |
| Day^2^ x Age _middle_ | 1.128 | .529 | 2.132 | .033 |
| Day^2^ x Age _old_ | .395 | .892 | .443 | .658 |
| Day^3^ x Age _middle_ | -.663 | .361 | -1.839 | .066 |
| Day^3^ x Age _old_ | .363 | .608 | .597 | .551 |
| Negative affect |  |  |  |  |
| Intercept | 5.346 | .017 | 308.215 | .000 |
| Region | .038 | .014 | 2.761 | .006 |
| Gender | -.296 | .009 | -31.812 | .000 |
| Age _middle_ | .272 | .027 | 10.173 | .000 |
| Age _old_ | -.259 | .044 | -5.875 | .000 |
| Day | 1.921 | .009 | 12.910 | .000 |
| Day^2^ | -5.910 | .360 | -16.401 | .000 |
| Day^3^ | 4.696 | .244 | 19.238 | .000 |
| Day x Age _middle_ | .083 | .231 | .358 | .720 |
| Day x Age _old_ | -1.471 | .390 | -3.774 | .000 |
| Day^2^ x Age _middle_ | .187 | .562 | .333 | .739 |
| Day^2^ x Age _old_ | 4.838 | .948 | 5.101 | .000 |
| Day^3^ x Age _middle_ | -.399 | .383 | -1.043 | .297 |
| Day^3^ x Age _old_ | -3.924 | .646 | -6.074 | .000 |
| Life satisfaction |  |  |  |  |
| Intercept | 5.987 | .018 | 340.614 | .000 |
| Region | -.063 | .014 | -4.405 | .000 |
| Gender | .326 | .010 | 33.849 | .000 |
| Age _middle_ | -.161 | .027 | -5.949 | .000 |
| Age _old_ | .140 | .044 | 3.147 | .002 |
| Day | -.978 | .151 | -6.494 | .000 |
| Day^2^ | 3.269 | .365 | 8.947 | .000 |
| Day^3^ | -2.911 | .248 | -11.747 | .000 |
| Day x Age _middle_ | -.554 | .234 | -2.366 | .018 |
| Day x Age _old_ | .509 | .392 | -1.296 | .195 |
| Day^2^ x Age _middle_ | 2.091 | .569 | 3.674 | .000 |
| Day^2^ x Age _old_ | .947 | .956 | .990 | .322 |
| Day^3^ x Age _middle_ | -1.387 | .388 | -3.574 | .000 |
| Day^3^ x Age _old_ | -.047 | .652 | -.072 | .943 |
| Life meaning |  |  |  |  |
| Intercept | 5.538 | .019 | 288.938 | .000 |
| Region | -.097 | .016 | -6.141 | .000 |
| Gender | .410 | .011 | 38.547 | .000 |
| Age _middle_ | .091 | .030 | 3.094 | .000 |
| Age _old_ | .445 | .048 | 9.210 | .002 |
| Day | -.848 | .164 | -5.169 | .000 |
| Day^2^ | 2.305 | .398 | 5.785 | .000 |
| Day^3^ | -2.179 | .270 | -8.057 | .000 |
| Day x Age _middle_ | -.769 | .255 | -3.018 | .003 |
| Day x Age _old_ | -.676 | .426 | -1.587 | .113 |
| Day^2^ x Age _middle_ | 2.988 | .621 | 4.814 | .000 |
| Day^2^ x Age _old_ | 1.630 | 1.038 | 1.570 | .116 |
| Day^3^ x Age _middle_ | -2.050 | .423 | -4.843 | .000 |
| Day^3^ x Age _old_ | -.494 | .708 | -.698 | .485 |
| *Note.* Day was rescaled to the maximum value of 1. Each age group represented in the age variable was coded 1 and the other two groups were 0 (e.g., Age _middle_ = 1, Age _young_ and Age _old_ = 0). Region and Gender were dummy coded (Daegu-Gyeongbuk = 1, Other regions =0; Male = 1, Female = 0). | | | | |
