# Supplementary material for: How COVID-19 affected mental well-being: An 11- week trajectories of daily well-being of Koreans amidst COVID-19 by age, gender and region
Source: PLoS One. 2021 Apr 23;16(4):e0250252. doi: 10.1371/journal.pone.0250252 (PMC8064534; doi:10.1371/journal.pone.0250252)
Supplement: S13 Table — (DOCX) [file pone.0250252.s015.docx]

| **S13 Table.** | | | | | |
| --- | --- | --- | --- | --- | --- |
| *The Results for Model Comparison between a Baseline model and a Day by Gender Interaction Model for Each Well-being Measure* | | | | | |
| Model | AIC | BIC | Log likelihood | $\chi^{2}$(df) | *p* |
| Well-being index |  |  |  |  |  |
| Cubic model | 1963581 | 1963692 | -981781 |  |  |
| Interaction model | 1963482 | 1963627 | -981728 | 105.170 (3) | .000 |
| Positive affect (PA) |  |  |  |  |  |
| Cubic model | 2098814 | 2098925 | -1049397 |  |  |
| Interaction model | 2098720 | 2098865 | -1049347 | 99.260 (3) | .000 |
| Negative affect (NA) |  |  |  |  |  |
| Cubic model | 2154839 | 2154950 | -1077409 |  |  |
| Interaction model | 2154775 | 2154919 | -1077375 | 69.609 (3) | .000 |
| Life satisfaction |  |  |  |  |  |
| Cubic model | 2174099 | 2174210 | -1087039 |  |  |
| Interaction model | 2174030 | 2174174 | -1087002 | 75.099 (3) | .000 |
| Life meaning |  |  |  |  |  |
| Cubic model | 2263573 | 2263684 | -1131776 |  |  |
| Interaction model | 2263521 | 2263665 | -1131747 | 58.045 (3) | .000 |
| Bored |  |  |  |  |  |
| Linear model | 2357367 | 2357455 | -1178675 |  |  |
| Interaction model | 2357355 | 2357455 | -1178669 | 13.653 (1) | .000 |
| Annoyed |  |  |  |  |  |
| Cubic model | 2381797 | 2381908 | -1190888 |  |  |
| Interaction model | 2381707 | 2381852 | -1190841 | 95.759 (3) | .000 |
| Depressed |  |  |  |  |  |
| Cubic model | 2378206 | 2378317 | -1189093 |  |  |
| Interaction model | 2378142 | 2378286 | -1189058 | 70.032 (3) | .000 |
| Anxious |  |  |  |  |  |
| Cubic model | 2405485 | 2405596 | -1202733 |  |  |
| Interaction model | 2405459 | 2405604 | -1202717 | 31.697 (3) | .000 |
| Stress |  |  |  |  |  |
| Cubic model | 2289050 | 2289161 | -1144515 |  |  |
| Interaction model | 2289010 | 2289155 | -1144492 | 45.992 (3) | .000 |
| Happy |  |  |  |  |  |
| Cubic model | 2197599 | 2197710 | -1098790 |  |  |
| Interaction model | 2197489 | 2197634 | -1098732 | 115.810 (3) | .000 |
| Joyful |  |  |  |  |  |
| Linear model | 2207245 | 2207334 | -1103615 |  |  |
| Interaction model | 2207198 | 2207298 | -1103590 | 48.664 (1) | .000 |
| Relaxed |  |  |  |  |  |
| Cubic model | 2274761 | 2274872 | -1137371 |  |  |
| Interaction model | 2274709 | 2274853 | -1137341 | 58.483 (3) | .000 |
| *Note.* Day was rescaled to have a range from 0 to 1. Each age group represented in the age variable was coded 1 and the other two groups were 0 (e.g., Age _middle_ = 1, Age _young_ and Age _old_ = 0). Region and Gender were dummy coded (Daegu-Gyeongbuk = 1, Other regions =0; Male = 1, Female = 0). | | | | | |
