# Supplementary material for: How COVID-19 affected mental well-being: An 11- week trajectories of daily well-being of Koreans amidst COVID-19 by age, gender and region
Source: PLoS One. 2021 Apr 23;16(4):e0250252. doi: 10.1371/journal.pone.0250252 (PMC8064534; doi:10.1371/journal.pone.0250252)
Supplement: S14 Table — (DOCX) [file pone.0250252.s016.docx]

| **S14 Table.**  *Results for Examining Day by Gender Interaction on Various Well-being Measures including Well-being Index, Positive Affect (PA), Negative Affect (NA), Life Satisfaction, and Life meaning* | | | | |
| --- | --- | --- | --- | --- |
| Predictor | Coefficient | *SE* | *t* | *p* |
| Well-being index |  |  |  |  |
| Intercept | 5.360 | .011 | 467.382 | .000 |
| Region | -.056 | .012 | -4.643 | .000 |
| Gender | .278 | .027 | 10.236 | .000 |
| Age _middle_ | -.103 | .007 | -14.961 | .000 |
| Age _old_ | .334 | .012 | 28.204 | .000 |
| Day | -1.888 | .095 | -19.828 | .000 |
| Day^2^ | 5.535 | .233 | 23.780 | .000 |
| Day^3^ | -4.312 | .159 | -27.141 | .000 |
| Day x Gender | 1.320 | .229 | 5.771 | .000 |
| Day^2^ x Gender | -4.049 | .552 | -7.339 | .000 |
| Day^3^ x Gender | 2.881 | .375 | 7.678 | .000 |
| Positive affect (PA) |  |  |  |  |
| Intercept | 5.686 | .013 | 422.907 | .000 |
| Region | -.042 | .013 | -3.201 | .001 |
| Gender | .247 | .032 | 7.818 | .000 |
| Age _middle_ | -.011 | .008 | -1.492 | .136 |
| Age _old_ | .282 | .013 | 21.697 | .000 |
| Day | -1.712 | .113 | -15.189 | .000 |
| Day^2^ | 5.171 | .274 | 18.843 | .000 |
| Day^3^ | -4.089 | .187 | -21.892 | .000 |
| Day x Gender | 1.279 | .267 | 4.783 | .000 |
| Day^2^ x Gender | -3.874 | .643 | -6.028 | .000 |
| Day^3^ x Gender | 2.671 | .436 | 6.125 | .000 |
| Negative affect (NA) |  |  |  |  |
| Intercept | 5.343 | .014 | 374.507 | .000 |
| Region | .038 | .014 | 2.750 | .006 |
| Gender | -.251 | .034 | -7.477 | .000 |
| Age _middle_ | .274 | .008 | 34.241 | .000 |
| Age _old_ | -.362 | .014 | -26.420 | .000 |
| Day | 2.126 | .120 | 17.750 | .000 |
| Day^2^ | -6.320 | .291 | -21.690 | .000 |
| Day^3^ | 4.849 | .198 | 24.460 | .000 |
| Day x Gender | -1.372 | .284 | -4.837 | .000 |
| Day^2^ x Gender | 4.252 | .681 | 6.240 | .000 |
| Day^3^ x Gender | -3.069 | .462 | -6.638 | .000 |
| Life satisfaction |  |  |  |  |
| Intercept | 5.958 | .014 | 411.913 | .000 |
| Region | -.064 | .014 | -4.444 | .000 |
| Gender | .335 | .034 | 9.832 | .000 |
| Age _middle_ | -.091 | .008 | -10.966 | .000 |
| Age _old_ | .184 | .014 | 12.959 | .000 |
| Day | -1.500 | .121 | -12.387 | .000 |
| Day^2^ | 4.975 | .295 | 16.865 | .000 |
| Day^3^ | -4.025 | .201 | -20.039 | .000 |
| Day x Gender | .983 | .288 | 3.415 | .001 |
| Day^2^ x Gender | -3.278 | .692 | -4.734 | .000 |
| Day^3^ x Gender | 2.343 | .470 | 4.983 | .000 |
| Life meaning |  |  |  |  |
| Intercept | 5.503 | .016 | 348.787 | .000 |
| Region | -.098 | .016 | -6.190 | .000 |
| Gender | .382 | .037 | 10.262 | .000 |
| Age _middle_ | .185 | .009 | 20.230 | .000 |
| Age _old_ | .519 | .016 | 33.080 | .000 |
| Day | -1.525 | .132 | -11.569 | .000 |
| Day^2^ | 4.554 | .321 | 14.171 | .000 |
| Day^3^ | -3.684 | .219 | -16.823 | .000 |
| Day x Gender | 1.261 | .314 | 4.014 | .000 |
| Day^2^ x Gender | -3.899 | .756 | -5.155 | .000 |
| Day^3^ x Gender | 2.763 | .514 | 5.378 | .000 |
| *Note.* Day was rescaled to the maximum value of 1. Each age group represented in the age variable was coded 1 and the other two groups were 0 (e.g., Age _middle_ = 1, Age _young_ and Age _old_ = 0). Region and Gender were dummy coded (Daegu-Gyeongbuk = 1, Other regions =0; Male = 1, Female = 0). | | | | |
