# Supplementary material for: How COVID-19 affected mental well-being: An 11- week trajectories of daily well-being of Koreans amidst COVID-19 by age, gender and region
Source: PLoS One. 2021 Apr 23;16(4):e0250252. doi: 10.1371/journal.pone.0250252 (PMC8064534; doi:10.1371/journal.pone.0250252)
Supplement: S15 Table — (DOCX) [file pone.0250252.s017.docx]

| **S15 Table.**  *Results for Examining Day by Gender Interaction on Negative Emotion Measures* | | | | |
| --- | --- | --- | --- | --- |
| Predictor | Coefficient | *SE* | *t* | *p* |
| Bored |  |  |  |  |
| Intercept | 5.385 | .010 | 9.016 | .000 |
| Region | -.273 | .020 | 3.522 | .000 |
| Gender | .155 | .017 | 9.393 | .000 |
| Age _middle_ | -.126 | .010 | 3.226 | .000 |
| Age _old_ | -.729 | .016 | 4.795 | .000 |
| Day | .644 | .015 | 3.944 | .000 |
| Day x Gender | .125 | .034 | 3.695 | .000 |
| Annoyed |  |  |  |  |
| Intercept | 4.330 | .018 | 239.337 | .000 |
| Region | .056 | .017 | 3.261 | .001 |
| Gender | -.206 | .042 | -4.853 | .000 |
| Age _middle_ | .564 | .010 | 56.899 | .000 |
| Age _old_ | .038 | .017 | 2.227 | .026 |
| Day | 3.296 | .152 | 21.635 | .000 |
| Day^2^ | -9.958 | .370 | -26.915 | .000 |
| Day^3^ | 7.699 | .251 | 30.619 | .000 |
| Day x Gender | -1.928 | .359 | -5.369 | .000 |
| Day^2^ x Gender | 6.237 | .861 | 7.240 | .000 |
| Day^3^ x Gender | -4.624 | .584 | -7.919 | .000 |
| Depressed |  |  |  |  |
| Intercept | 4.479 | .018 | 248.972 | .000 |
| Region | .033 | .017 | 1.900 | .057 |
| Gender | -.451 | .042 | -10.669 | .000 |
| Age _middle_ | .201 | .010 | 20.255 | .000 |
| Age _old_ | -.303 | .017 | -17.852 | .000 |
| Day | 2.635 | .151 | 17.418 | .000 |
| Day^2^ | -8.146 | .368 | -22.155 | .000 |
| Day^3^ | 6.351 | .250 | 25.401 | .000 |
| Day x Gender | -1.395 | .357 | -3.906 | .000 |
| Day^2^ x Gender | 4.874 | .858 | 5.683 | .000 |
| Day^3^ x Gender | -3.732 | .581 | -6.418 | .000 |
| Anxious |  |  |  |  |
| Intercept | 4.635 | .018 | 250.951 | .000 |
| Region | .021 | .018 | 1.169 | .242 |
| Gender | -.357 | .043 | -8.218 | .000 |
| Age _middle_ | .131 | .010 | 12.721 | .000 |
| Age _old_ | -.415 | .018 | -23.645 | .000 |
| Day | 2.817 | .155 | 18.148 | .000 |
| Day^2^ | -7.451 | .377 | -19.743 | .000 |
| Day^3^ | 5.308 | .257 | 20.678 | .000 |
| Day x Gender | -1.318 | .367 | -3.592 | .000 |
| Day^2^ x Gender | 4.026 | .881 | 4.569 | .000 |
| Day^3^ x Gender | -2.930 | .598 | -4.904 | .000 |
| Stress |  |  |  |  |
| Intercept | 5.961 | .016 | 361.490 | .000 |
| Region | .011 | .015 | 0.707 | .480 |
| Gender | -.234 | .039 | -6.038 | .000 |
| Age _middle_ | .360 | .009 | 40.207 | .000 |
| Age _old_ | -.369 | .015 | -24.173 | .000 |
| Day | 1.874 | .139 | 13.477 | .000 |
| Day^2^ | -6.021 | .337 | -17.843 | .000 |
| Day^3^ | 4.649 | .229 | 20.281 | .000 |
| Day x Gender | -1.102 | .327 | -3.368 | .001 |
| Day^2^ x Gender | 3.555 | .784 | 4.533 | .000 |
| Day^3^ x Gender | -2.578 | .532 | -4.850 | .000 |
| *Note.* Day was rescaled to the maximum value of 1. Each age group represented in the age variable was coded 1 and the other two groups were 0 (e.g., Age _middle_ = 1, Age _young_ and Age _old_ = 0). Region and Gender were dummy coded (Daegu-Gyeongbuk = 1, Other regions =0; Male = 1, Female = 0). | | | | |
