# Supplementary material for: How COVID-19 affected mental well-being: An 11- week trajectories of daily well-being of Koreans amidst COVID-19 by age, gender and region
Source: PLoS One. 2021 Apr 23;16(4):e0250252. doi: 10.1371/journal.pone.0250252 (PMC8064534; doi:10.1371/journal.pone.0250252)
Supplement: S16 Table — (DOCX) [file pone.0250252.s018.docx]

| **S16 Table.**  *Results for Examining Day by Gender Interaction on Positive Emotion Measures* | | | | |
| --- | --- | --- | --- | --- |
| Predictor | Coefficient | *SE* | *t* | *p* |
| Happy |  |  |  |  |
| Intercept | 5.914 | .015 | 398.161 | .000 |
| Region | -.053 | .015 | -3.603 | .000 |
| Gender | .247 | .035 | 7.059 | .000 |
| Age _middle_ | .012 | .008 | 1.471 | .141 |
| Age _old_ | .286 | .014 | 19.818 | .000 |
| Day | -1.481 | .124 | -11.901 | .000 |
| Day^2^ | 4.887 | .303 | 16.127 | .000 |
| Day^3^ | -4.019 | .206 | -19.483 | .000 |
| Day x Gender | 1.605 | .295 | 5.431 | .000 |
| Day^2^ x Gender | -4.806 | .710 | -6.766 | .000 |
| Day^3^ x Gender | 3.311 | .482 | 6.868 | .000 |
| Joyful |  |  |  |  |
| Intercept | 5.379 | .009 | 8.063 | .000 |
| Region | -.048 | .014 | 3.421 | .001 |
| Gender | .429 | .017 | 4.814 | .000 |
| Age _middle_ | .015 | .008 | 1.845 | .065 |
| Age _old_ | .235 | .014 | 6.855 | .000 |
| Day | -.281 | .013 | 2.327 | .000 |
| Day x Gender | -.202 | .029 | 6.976 | .000 |
| Relaxed |  |  |  |  |
| Intercept | 5.722 | .016 | 352.422 | .000 |
| Region | -.025 | .015 | -1.658 | .097 |
| Gender | .201 | .038 | 5.286 | .000 |
| Age _middle_ | -.064 | .009 | -7.278 | .000 |
| Age _old_ | .335 | .015 | 22.139 | .000 |
| Day | -2.258 | .137 | -16.505 | .000 |
| Day^2^ | 6.914 | .332 | 20.816 | .000 |
| Day^3^ | -5.362 | .226 | -23.759 | .000 |
| Day x Gender | 1.023 | .322 | 3.174 | .002 |
| Day^2^ x Gender | -3.414 | .773 | -4.419 | .000 |
| Day^3^ x Gender | 2.464 | .524 | 4.704 | .000 |
| *Note.* Day was rescaled to the maximum value of 1. Each age group represented in the age variable was coded 1 and the other two groups were 0 (e.g., Age _middle_ = 1, Age _young_ and Age _old_ = 0). Region and Gender were dummy coded (Daegu-Gyeongbuk = 1, Other regions =0; Male = 1, Female = 0). | | | | |
