# Supplementary material for: How COVID-19 affected mental well-being: An 11- week trajectories of daily well-being of Koreans amidst COVID-19 by age, gender and region
Source: PLoS One. 2021 Apr 23;16(4):e0250252. doi: 10.1371/journal.pone.0250252 (PMC8064534; doi:10.1371/journal.pone.0250252)
Supplement: S17 Table — (DOCX) [file pone.0250252.s019.docx]

| **S17 Table.** | | | | |
| --- | --- | --- | --- | --- |
| *Results for the Multilevel Analyses on the Well-being Index using Subsamples Providing two or more Daily Well-being Responses* | | | | |
| Predictor | *Coefficient* | *SE* | *t* | *p* |
| Well-being index | |  |  |  |
| Intercept | 5.481 | 0.017 | 318.509 | 0.000 |
| Region | 0.073 | 0.028 | -2.618 | 0.009 |
| Gender | 0.233 | 0.021 | 11.208 | 0.000 |
| Age _middle_ | -0.239 | 0.016 | -14.677 | 0.000 |
| Age _old_ | 0.274 | 0.024 | 11.497 | 0.000 |
| Day | -1.341 | 0.123 | -10.903 | 0.000 |
| Day^2^ | 3.721 | 0.309 | 12.063 | 0.000 |
| Day^3^ | -2.894 | 0.214 | -13.507 | 0.000 |
| *Note.* Day was rescaled to the maximum value of 1. Each age group represented in the age variable was coded 1 and the other two groups were 0 (e.g., Age _middle_ = 1, Age _young_ and Age _old_ = 0). Region and Gender were dummy coded (Daegu-Gyeongbuk = 1, Other regions =0; Male = 1, Female = 0). | | | | |
